# Global, regional, and national time trends in incidence, prevalence, years lived with disability for uterine fibroids, 1990–2019: an age-period-cohort analysis for the global burden of disease 2019 study

**DOI:** 10.1186/s12889-023-15765-x

**Published:** 2023-05-19

**Authors:** Zheng Lou, Yizhou Huang, Shuting Li, Zhou Luo, Chunming Li, Ketan Chu, Tao Zhang, Peige Song, Jianhong Zhou

**Affiliations:** 1 Department of Gynecology, Women’s Hospital, Zhejiang University School of Medicine, Hangzhou, China; 2 School of Public Health, Zhejiang University School of Medicine, Zhejiang University, Hangzhou, China; 3 School of Public Health and Women’s Hospital, Zhejiang University School of Medicine, Hangzhou, China

**Keywords:** Uterine fibroid, Global burden health, Age-period-cohort model, Epidemiology, Incidence, Prevalence, Years lived with disability

## Abstract

**Background:**

Uterine fibroids are the most common benign neoplasm of the uterus and a major source of morbidity for women. We report an overview of trends in uterine fibroids of incidence rate, prevalence rate, years lived with disability (YLDs) rate in 204 countries and territories over the past 30 years and associations with age, period, and birth cohort.

**Methods:**

The incident case, incidence rate, age-standardized rate (ASR) for incidence, prevalent case, prevalence rate, ASR for prevalence, number of YLDs, YLD rate, and ASR for YLDs were derived from the Global Burden of Disease 2019 (GBD 2019) study. We utilized an age-period-cohort (APC) model to estimate overall annual percentage changes in the rate of incidence, prevalence, and YLDs (net drifts), annual percentage changes from 10 to 14 years to 65–69 years (local drifts), period and cohort relative risks (period/cohort effects) between 1990 and 2019.

**Results:**

Globally, the incident cases, prevalent cases, and the number of YLDs of uterine fibroids increased from 1990 to 2019 with the growth of 67.07%, 78.82% and 77.34%, respectively. High Socio-demographic Index (SDI) and high-middle SDI quintiles with decreasing trends (net drift < 0.0%), and increasing trends (net drift > 0.0%) were observed in middle SDI, low-middle SDI, and low SDI quintiles in annual percentage change of incidence rate, prevalence rate and YLDs rate over the past 30 years. There were 186 countries and territories that showed an increasing trend in incidence rate, 183 showed an increasing trend in prevalence rate and 174 showed an increasing trend in YLDs rate. Moreover, the effects of age on uterine fibroids increased with age and peaked at 35–44 years and then declined with advancing age. Both the period and cohort effects on uterine fibroids showed increasing trend in middle SDI, low-middle SDI and low SDI quintiles in recent 15 years and birth cohort later than 1965.

**Conclusions:**

The global burden of uterine fibroids is becoming more serious in middle SDI, low-middle SDI and low SDI quintiles. Raising awareness of uterine fibroids, increasing medical investment and improving levels of medical care are necessary to reduce future burden.

**Supplementary Information:**

The online version contains supplementary material available at 10.1186/s12889-023-15765-x.

## Background

Uterine fibroids are the most common benign neoplasm of the uterus and a major source of morbidity for women of reproductive age, affecting up to 68.6% women [1, 2]. Premenopausal age, black race, nulliparity, and time since last birth are the main identified risk factors [3]. Approximately 30% of fibroid women present with severe symptoms, including abnormal uterine bleeding, iron-deficiency anemia, infertility, pelvic pain, back pain and urinary symptoms (such as frequent urination, nocturia or urinary retention) or gastrointestinal symptom (such as diarrhea or constipation) that require intervention [4]. Options for symptomatic fibroid treatment include expectant, medical, interventional radiology procedures and surgical management [5]. Hysterectomy remains the sole definitive treatment for symptomatic women [6]. Globally, uterine fibroids contribute to at least one-third and up to half of all hysterectomies, removing the possibility of childbearing and having long consequences for general health [7–9].

High prevalence of uterine fibroids has a profound effect on healthcare costs globally. According to estimates, costs related to uterine fibroid are up to 34.4 billion dollars annually in the United States, 348 million in Germany, 120 million in France, and 86 million in England, surpassing the other two common cancers in women, breast cancer and ovarian cancer [10, 11]. In addition to the direct health care expenses, indirect costs due to lost income from time off work and disability due to uterine fibroid are estimated to be 1.6 to 17.2 billion dollars annually [12, 13]. Uterine fibroids are also related to infertility and other pregnancy complications, which can last for at least ten years of treatment and account for 4–23% of the annual costs [13, 14].

However, there are several challenges in understanding the epidemiology of uterine fibroids globally. The first issue is that most women with uterine fibroids are asymptomatic and the uterine fibroids are discovered by accident during a routine gynecologic examination or procedures [15]. The large number of undetected uterine fibroids creates a significant bias in epidemiological data. Another important source of confusion is that only a few studies have been conducted based on a handful of countries to explore the incidence or prevalence of uterine fibroids to date [16–18]. It is still noteworthy that different types of research, diagnosis methods, and racial/ethnic demographic factors affect uterine fibroids incidence and prevalence in different countries and studies [19]. Besides, studies designed to examine the epidemiologic trend of uterine fibroids are even more scant. Given the considerable impact on physical, social and public health, understanding global variations in the burden of uterine fibroids is pivotal. It can be used to identify factors contributing to these variations, and to make sensible decisions regarding the allocation of resources for disease screening and management.

As a consequence, this study aims to investigate the long-term epidemiologic trends of uterine fibroids globally from 1990 to 2019 by utilizing data from the Global Burden of Disease 2019 (GBD 2019) and the age-period-cohort (APC) framework to examine independent effects of age, period, birth cohort. Findings from this study could provide certain enlightenment for the resource allocation of vulnerable groups.

## Materials and methods

### Date source and case definition

GBD 2019 provides a total of 369 diseases and injuries in 204 countries and terries between 1990 and 2019 with not only population estimates, but also a comprehensive assessment of incidence, prevalence, mortality, years of life lost, years lived with disability (YLDs) which were the number of years lived with a disability multiplied with a disability weight reflecting severity of disability, and disability-adjusted life years [20, 21]. To estimate the burdern of uterine fibroids globally, the GBD used 321 data input sources that corresponded to literature data, claims data, and hospital administrative data. The Global Health Data Exchange (GHDx) Data Input Sources Tool (http://ghdx.healthdata.org/gbd-2019/data-input-sources) provides a detailed list and information about the data input sources. Uterine fibroids, also knew as uterine myomas or leiomyomas, are non-cancerous tumors that develop from the muscle tissue of the uterus [22]. For this study, uterine fibroids cases for incidence, prevalence, and YLDs data were identified as codes D25-D26.9, D28.2 according to the tenth of International Classification of Diseases (ICD) codes, (ICD-10) [22].

### Estimation of uterine fibroids in incidence, prevalence, and YLDs

The modeling framework and a detailed flowchart including specific codes for uterine fibroids estimation in the GBD are available at https://ghdx.healthdata.org/gbd-2019/code/nonfatal-13. The modeling steps included (1) compiling data sources through data identification and extraction; (2) data adjustment; (3) estimation of incidence/prevalence/YLDs by using DisMod-MR 2.1 [22].

### Reporting standards

To describe the uterine fibroids globally, we used data publicly available online at https://vizhub.healthdata.org/gbd-results/. We collected the incident case, incidence rate, age-standardized rate (ASR) for incidence, prevalent case, prevalence rate, ASR for prevalence, number of YLDs, YLD rate, and ASR for YLDs of uterine fibroids from 1990 to 2019, from 10 years to 69 years old according to 204 countries or territories and 5 Socio-demographic Index (SDI) quintiles, reported with the 95% uncertainty intervals (UIs). The 95% UIs represents the 2.5th and 97.5th percentiles of distribution of 1000 random draws conducted at each modeling stage [22]. All countries were categorized into one of five-SDI quintiles (i.e., high, high-middle, middle, low-middle and low) based on 2019 SDI values. SDI (range from 0 to 1) was used as an indicator for each country to estimate the composition of income per capita, average years of schooling, and fertility rate in females under 25 years old. A higher SDI indicates a higher socioeconomic level [21].

### Age-period-cohort modelling analysis of incidence rate, prevalence rate and YLDs rate data

The APC model is used in this study to analyze the underlying trends in incidence rate, prevalence rate, and YLDs rate of uterine fibroids by age, period, and birth cohort [23]. By extending beyond traditional epidemiological analysis, APC model unravels the contributions of biological factors associated with aging, as well as technological and social factors, to disease trends [24]. The age effect refers to the change in disease with age, one of the most important factors in causing disease. The period effect is reflection of changes in social, cultural, economic or physical environments over time that affect all age groups simultaneously. The cohort effect can be defined as the change in the characteristics of groups with the same birth year [25]. Generally, the APC model.

can be expressed as follows:

Y = log (*M*) = µ + α(age)_*i*_ + β(period)_*j*_ + γ(cohort)_*k*_ + ε.

where *M* denotes the incidence rate/ prevalence rate/ YLDs rate of uterine fibroids, µ and ε are defined as the intercept and random error, and α(age)_*i*_, β(period)_*j*_, γ(cohort)_*k*_ denote the effects of age group α, time period β, and birth cohort γ, respectively.

Extracted from GBD 2019, the incidence rate, prevalence rate and YLDs rate estimates for uterine fibroids and population data of each region and country were used as data inputs in the APC model. For APC analysis, we arranged the data into consecutive age group with five-year age intervals from 10 to 14 years to 65–69 years and successive five-year period: from 1990 to 1994 (median 1992) to 2015–2019 (median 2017), with 2000–2004 as the reference period. In the meanwhile, we arranged 17 partially overlapping ten-year birth cohort, from 1921 to 1929 (the 1925 cohort) to 2001–2009 (the 2005 cohort) as referenced by the 1961–1969 (the 1965 cohort) birth cohort. The longitudinal age curve indicates the fitted longitudinal age-specific rates adjusted for the period deviation in the reference cohort. The relative risks (RRs) of period and cohort are calculated by comparing age-specific rates in each period and each cohort to a reference group, respectively. There are two significant parameters in APC models: net drift and local drift. Net drift refers to the overall average annual percentage change of the expected age-adjusted rates over time, based on the log-linear trend by period and birth cohort. The local drift represents the annual percentage change in the expected age-specific rates over time based on the log-linear trend of period and birth cohort for each age group [26]. The drift above 0.0% per year is considered an increasing trend and the drift lower than 0.0% annually shows a decreased trend in annual percentage change. Wald chi-squared test was used to test the significance of trend in annual percentage change. Two-sided statistical tests were performed, and *p*＜0.05 was considered significant. The analysis was conducted in R (version 4.1.2).

### Data disclosure statement

Data for this study is compiled from the GBD 2019 database, which does not contain any identifiable personal information. Informed consent waiver was reviewed and approved by the University of Washington Institutional Review Board.

## Results

### The burden of uterine fibroids at global and regional level

Table 1 shows the incident cases, ASR for incidence, prevalent cases, ASR for prevalence, number of YLDs, ASR for YLDs, as well as the net drift. Over the past 30 years, the incident cases increased from 5.77 million to 9.64 million, with growth of 67.07%. Globally in 2019, ASR for incidence was 241.18 (95% UI: 179.45 to 318.02) per 100,000 women, an 6.87% increased from 1990 and the net drift was − 0.04% (95% CI: -0.15 to 0.07) per year. The ASR for incidence in 2019 ranged from 218.56 (95% UI: 162.86 to 287.17) per 100,000 women in middle SDI region to 262.37 (95% UI: 196.04 to 344.26) per 100,000 women in high SDI region. Of the five-SDI regions, two regions (high SDI and high-middle SDI regions) had decreased trend for incidence rate from 1990 to 2019 where the largest reduction was in high-middle SDI region with the net drift − 0.54% (95% CI: -0.65 to -0.42) per year. Total of three regions (middle SDI, low-middle SDI and low SDI) showed an upward trend for incidence rate from 1990 to 2019 where the highest growth was in low-middle SDI regions with the net drift 0.51% (95% CI: 0.38 to 0.63) per year.

**Table 1:** The incidence, prevalence, years lived with disability and age-standardized rate of uterine fibroids in 1990 and 2019, and the temporal trends from 1990 to 2019 at global and regional level

Incidence					
	Cases in1990	ASR in 1990 (per 100 000 persons)	Cases in 2019	ASR in 2019 (per 100 000persons)	Net Drift (%, per year)
Global	5769658 (4274825–7634396)	225.67 (167.33-298.87)	9643336 (7178053–12714741)	241.18 (179.45-318.02)	-0.04 (-0.15-0.07)
High SDI	1148287 (850122–1510514)	258 (191.85–338.10)	1293913 (967684–1700772)	262.37 (196.04-344.26)	-0.30 (-0.63-0.02)
High-middle SDI	1597605 (1178845–2125199)	267.44 (198.61–353.70)	2016160 (1498310–2653759)	254.36 (189.78-333.77)	-0.54 (-0.65–0.42)
Middle SDI	1541213 (1141080–2060055)	187.02 (138.12-248.69)	2863366 (2136007–3771451)	218.56 (162.86-287.17)	0.20 (0.05–0.35)
Low-middle SDI	1039024 (772566–1383163)	214.77 (159.34-285.23)	2351345 (1740193–3122571)	260.21 (191.86–346.8)	0.51 (0.38–0.63)
Low SDI	440110 (329441–587476)	208.77 (154.92-278.63)	1113192 (828604–1476128)	227.28 (169.27-302.26)	0.23 (0.17–0.29)
Prevalence					
	Cases in1990	ASR in 1990 (per 100 000 persons)	Cases in 2019	ASR in 2019 (per 100 000persons)	Net Drift (%, per year)
Global	126409021 (97245515–163093351)	5379.82 (4120.48-6876.82)	226045773 (174804844–287321267)	5467.68 (4210.51-6975.18)	0.02 (-0.02-0.06)
High SDI	29764671 (23173210–37877444)	6442 (4988.57-8181.54)	37821797 (29529453–47240503)	6325.28 (4924.81-7943.16)	-0.14 (-0.27–0.02)
High-middle SDI	37534454 (28909262–47539653)	6449.56 (4966.03-8171.93)	51967864 (40360677–65888573)	5794.45 (4490.57-7358.55)	-0.41 (-0.45–0.36)
Middle SDI	30301756 (23158757–39568640)	4234.62 (3220.77-5483.1)	65466135 (50443124–83829589)	4790.48 (3690.02-6152.4)	0.35 (0.32–0.38)
Low-middle SDI	20336490 (15571404–26526175)	4815.58 (3654.24-6227.46)	49409116 (37679311–64021661)	5801.79 (4435.93-7508.02)	0.66 (0.63–0.69)
Low SDI	8399173 (6417455–10961070)	4688.25 (3546.56-6044.91)	21252674 (16205864–27779182)	5140.35 (3902.85-6634.41)	0.31 (0.29–0.32)
YLDs					
	Cases in1990	ASR in 1990 (per 100 000 persons)	Cases in 2019	ASR in 2019 (per 100 000persons)	Net Drift (%, per year)
Global	706034 (330340–1324679)	29.96 (14.05–56.24)	1252087 (590244–2326874)	30.32 (14.25–56.35)	0.01 (-0.02-0.05)
High SDI	162287 (74995–303965)	35.18 (16.24–66.13)	205183 (96426–386790)	34.5 (16.04–65.21)	-0.15 (-0.28–0.03)
High-middle SDI	206863 (95560–395851)	35.52 (16.41–68.14)	284911 (132407–537924)	31.88 (14.88–60.4)	-0.42 (-0.46–0.37)
Middle SDI	171533 (81451–320862)	23.83 (11.26–44.81)	362648 (170259–674632)	26.57 (12.5-49.39)	0.31 (0.28–0.34)
Low-middle SDI	116493 (55762–215905)	27.42 (13.23–50.72)	277265 (131448–519624)	32.48 (15.48–60.63)	0.62 (0.59–0.66)
Low SDI	48452 (23044–88794)	26.85 (12.92–49.43)	121368 (57592–226120)	29.14 (13.92-54)	0.29 (0.24–0.33)

**Note:** YLDs: years lived with disability; ASR: age-standardized rate

ASR for incidence/ prevalence/ YLDs is computed by direct standardization with global standard population in GBD 2019.

Net drifts are estimates derived from the age-period-cohort model and denotes overall annual percent change in incidence rate/ prevalence rate/ YLDs rate

Parenthesis for all GBD health estimate indicates 95% uncertainty intervals; parenthesis for net drift indicates 95% confidence intervals

Over the past 30 years, the prevalent cases increased from 126.41 million to 226.05 million, with growth of 78.82%. Globally in 2019, ASR for prevalence was 5467.68 (95% UI: 4210.51 to 6975.18) per 100,000 women, an 1.63% increased from 1990 and the net drift was 0.02% (95% CI: -0.02 to 0.06) per year. The ASR for prevalence in 2019 ranged from 4790.48 (95% UI: 3690.02 to 6152.4) per 100,000 women in middle SDI region to 6325.28 (95% UI: 4924.81 to 7943.16) per 100,000 women in high SDI region. Of the five-SDI regions, two regions (high SDI and high-middle SDI regions) had decreased trend for prevalence rate from 1990 to 2019 where the largest reduction was in high-middle SDI region with the net drift − 0.41% (95% CI: -0.45 to -0.36) per year. Total of three regions (middle SDI, low-middle SDI and low SDI) showed an upward trend of prevalence rate from 1990 to 2019 where the highest growth was in low-middle SDI regions with the net drift 0.66% (95% CI: 0.63 to 0.69) per year.

The number of YLDs increased from 0.71 million to 1.25 million with growth of 77.34% over the past 30 years. Globally in 2019, ASR for YLDs was 30.32 (95% UI: 14.25 to 56.35) per 100,000 women with an increase of 1.20% and the net drift was 0.01% (95% CI: -0.02 to 0.05) per year. The ASR for YLDs in 2019 ranged from 26.57 (95% UI: 12.5 to 49.39) per 100,000 women in middle SDI region to 34.5 (95% UI: 16.04 to 65.21) per 100,000 women in high SDI region. Of the five-SDI regions, two regions (high SDI and high-middle SDI regions) had decreased trend of YLDs rate from 1990 to 2019 where the largest reduction was in high-middle SDI region with the net drift − 0.42% (95% CI: -0.46 to -0.37) per year. Total of three regions (middle SDI, low-middle SDI and low SDI) showed an upward trend of YLDs rate from 1990 to 2019 where the highest growth was in low-middle SDI regions with the net drift 0.62% (95% CI: 0.59 to 0.66) per year.

### The burden of uterine fibroids at national level

Amongst 204 countries and territories, the ASR for incidence ranged from 81.98 to 667.14 per 100,000 women. Latvia (667.14 [95% UI: 492.30 to 884.54] per 100,000 women), Russian Federation (586.64 [95% UI: 434.96 to 771.37] per 100,000 women) and Ukraine (578.21 [95% UI: 427.17 to 766.78] per 100,000 women) had the highest ASR for incidence, while New Zealand (81.98 [95% UI: 62.13 to 104.51] per 100,000 women), Australia (86.13 [95% UI: 62.44 to 114.45] per 100,000 women) and Democratic People’s Republic of Korea (103.72 [95% UI: 75.68 to 138.13] per 100,000 women) had the lowest. Of the 204 countries and territories, 186 showed an increasing trend (net drift > 0.0% per year) in incidence rate. Brazil (net drift: 1.47% [95% CI: 1.38 to 1.56] per year), India (net drift: 0.89% [95% CI: 0.85 to 0.93] per year) and United States of America (net drift: 0.65% [95% CI: 0.58 to 0.73] per year) showed the largest increases. Poland (net drift: -0.59% [95% CI: -0.67 to -0.51] per year), New Zealand (net drift: -0.37% [95% CI: -0.42 to -0.33] per year) and United Kingdom (net drift: -0.33% [95% CI: -0.40 to -0.26] per year) had the most significant decrease (supplementary Figure S1, supplementary Table S1).

**Fig. 1:**
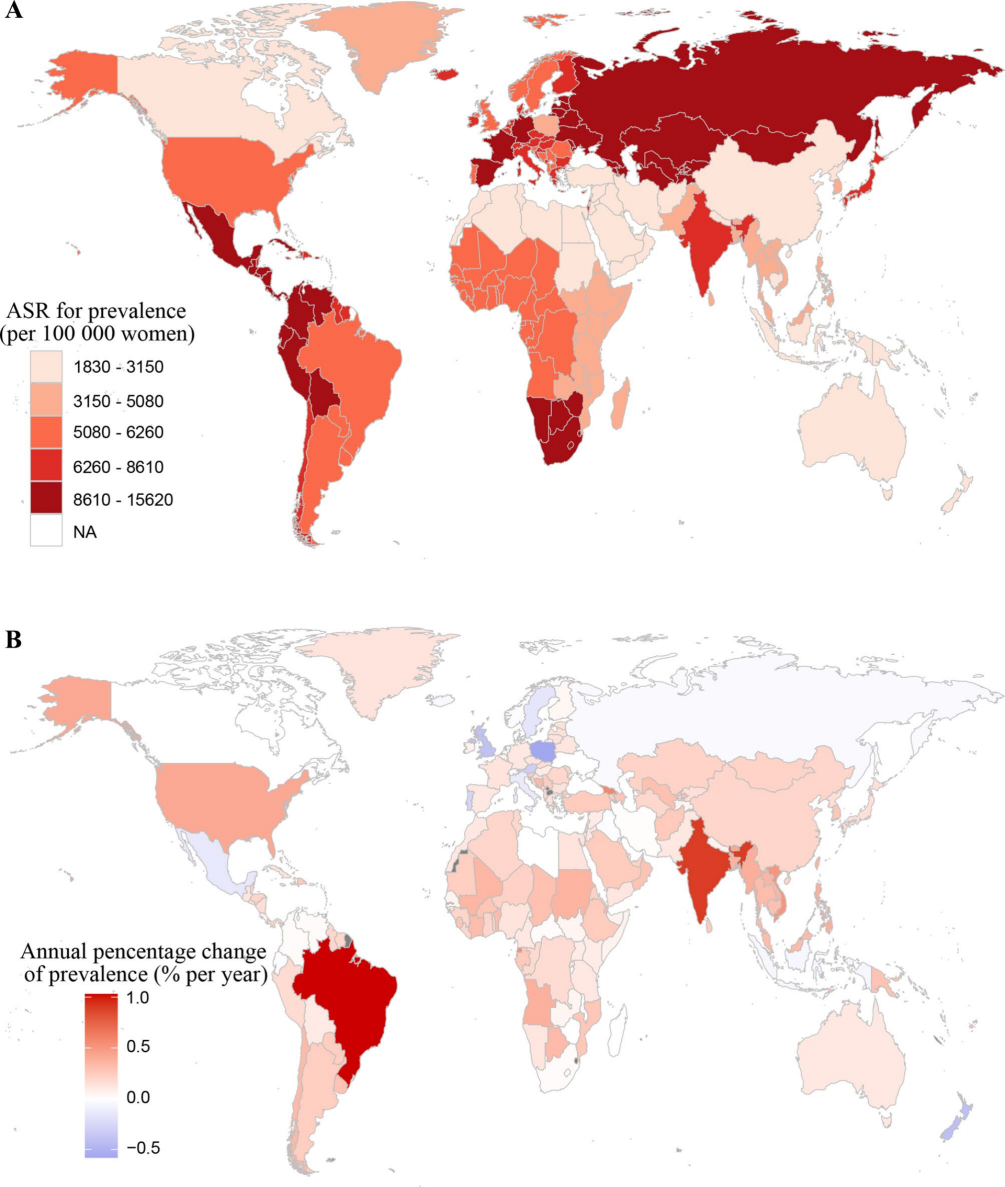
The global distribution of age-standardized rate for prevalence in 2019 **(A)** and net drift of prevalence rate during 1990 − 2019 **(B)** for uterine fibroids in 204 countries and territories

The ASR for prevalence also varied substantially by country in 2019 (from 1830.67 to 15612.81) per 100,000 women. Latvia (15612.81 [95% UI: 11803.14 to 20170.30] per 100,000 women), Russian Federation (13336.71 [95% UI: 10236.11 to 16959.87] per 100,000 women) and Estonia (13127.11 [95% UI: 9989.26 to 17172.97] per 100,000 women) had the highest ASR for prevalence. In contrast, New Zealand (1830.67 [95% UI: 1451.65 to 2274.51] per 100,000 women), Australia (1915.86 [95% UI: 1449.97 to 2499.94] per 100,000 women) and Democratic People’s Republic of Korea (2227.76 [95% UI: 1672.86 to 2904.66] per 100,000 women) showed the lowest. Of the 204 countries and territories, 183 showed an increasing trend (net drift > 0.0% per year) in prevalence rate. Brazil (1.03% [95% CI: 0.95 to 1.12] per year), India (0.90% [95% CI: 0.87 to 0.94] per year) and Georgia (0.58% [95% CI: 0.55 to 0.61] per year) showed the largest increasing trend. Poland (-0.58% [95% CI: -0.65 to -0.52] per year), New Zealand (-0.41% [95% CI: -0.47 to -0.36] per year) and United Kingdom (-0.40% [95% CI: -0.43 to -0.36] per year) had the most significant decrease in net drift (Fig. 1, supplementary Table S2).

The ASR for YLDs ranged from 9.99 to 85.77 per 100,000 women across the 204 countries and territories in 2019. Latvia (85.77 [95% UI: 39.64 to 163.35] per 100,000 women), Russian Federation (73.12 [95% UI: 33.61 to 141.63] per 100,000 women) and Estonia (72.03 [95% UI: 33.29 to 138.61] per 100,000 women) had the highest ASR for YLDs. In contrast, New Zealand (9.99 [95% UI: 4.67 to 19.00] per 100,000 women), Australia (10.49 [95% UI: 4.72 to 20.48] per 100,000 women) and Democratic People’s Republic of Korea (12.54 [95% UI: 5.86 to 23.39] per 100,000 women) showed the lowest. Of the 204 countries and territories, 174 showed an increasing trend (net drift > 0.0% per year) in YLDs rate. Brazil (0.99% [95% CI: 0.90 to 1.08] per year), India (0.85% [95% CI: 0.80 to 0.89] per year) and Georgia (0.56% [95% CI: 0.53 to 0.59] per year) showed the largest increasing trends. Poland (-0.59% [95% CI: -0.64 to -0.54] per year), United Kingdom (-0.40% [95% CI: -0.44 to -0.36] per year) and New Zealand (-0.40% [95% CI: -0.45 to -0.35] per year) had the most significant decrease in net drift (supplementary Figure S2, supplementary Table S3).

### Time trends in incidence rate, prevalence rate and YLDs rate of uterine fibroids across different age groups

**Fig. 2:**
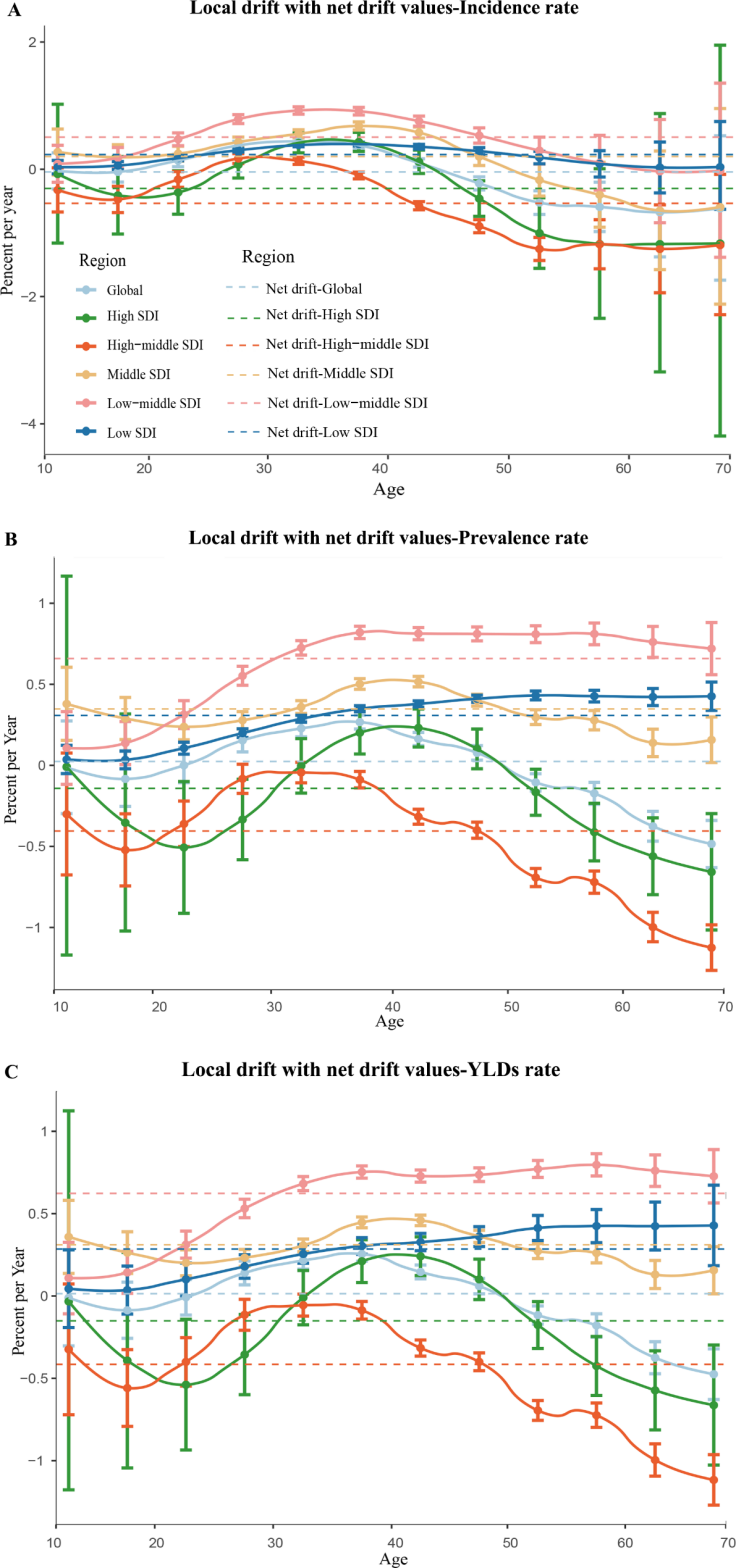
Local drift with net drift values for incidence rate **(A)**, prevalence rate **(B)**, years lived with disability rate **(C)** of uterine fibroids in global and five-SDI quintiles from 1990 to 2019. Note: A-C use the same set of legends

Figure 2 showed the annual percentage change in the incidence rate, prevalence rate and YLDs rate of uterine fibroids for each age group, from 10 to 14 years to 65–69 years in five-year intervals. Globally, the age group from 25 to 29 years to 45–49 years showed an increasing trend in incidence rate, prevalence rate and YLDs rate, with the highest trend in 35–39 age group (0.27% [95% CI: 0.22 to 0.31] per year; 0.27% [95% CI: 0.22 to 0.31] per year; 0.25% [95% CI: 0.21 to 0.30] per year). After age of 50, the declining trend attenuated with increasing age, with lowest in the oldest age group (-0.48% [95% CI: -0.64 to -0.32] per year; -0.49% [95% CI: -0.63 to -0.34] per year; -0.48% [95% CI: -0.63 to -0.32] per year). Of note, in middle SDI region, low-middle SDI region and low SDI region, uterine fibroids in prevalence rate and YLDs rate had increasing trends across almost all age groups.

### Age, period, and cohort effects on uterine fibroids incidence rate, prevalence rate and YLDs rate

**Fig. 3:**
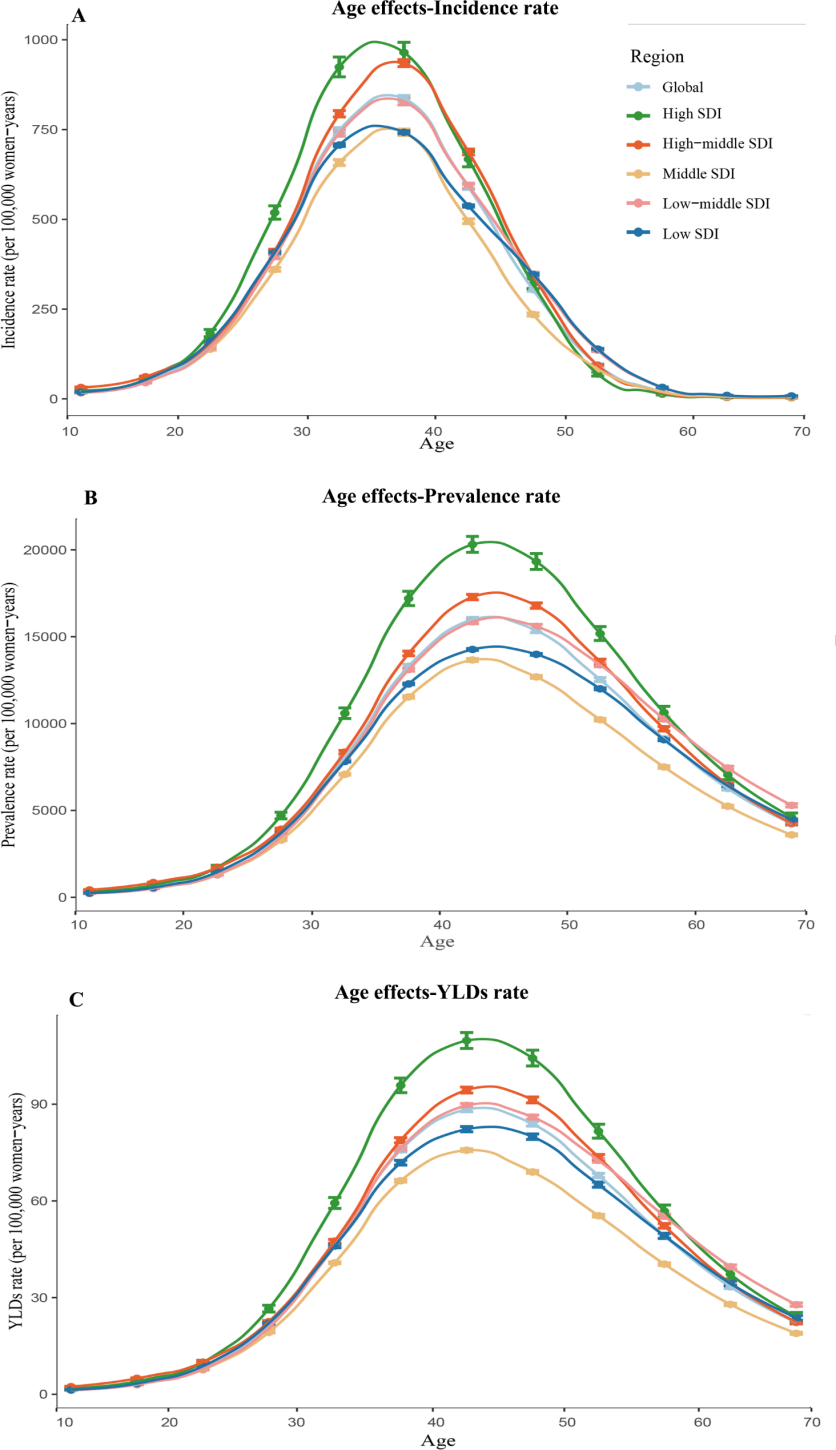
Parameter estimates of age effects on incidence rate(A), prevalence rate(B), years lived with disability rate(C) of uterine fibroids in global and five-SDI quintiles from 1990 to 2019. Note: A-C use the same set of legends

**Fig. 4:**
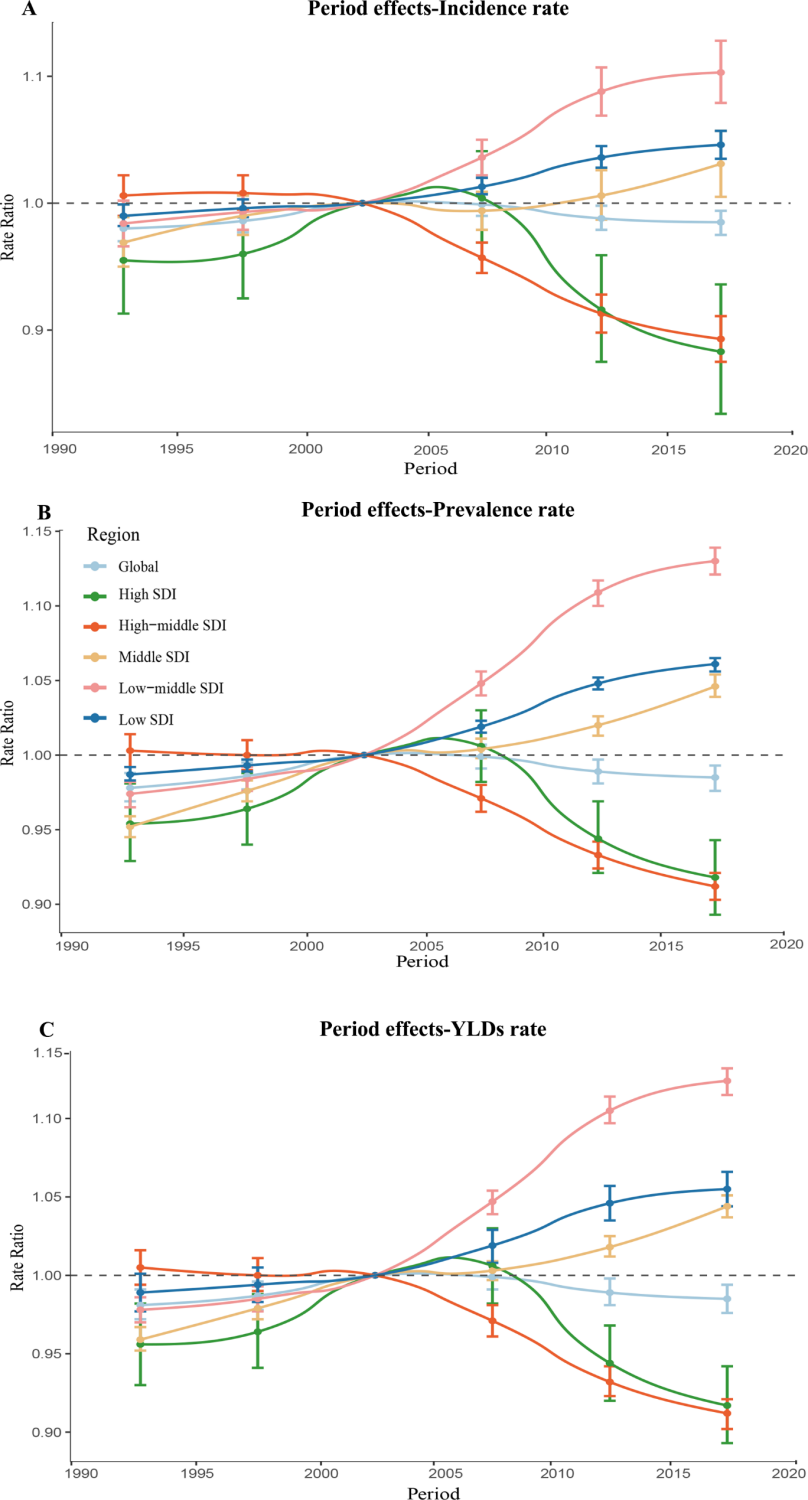
Parameter estimates of period effects on incidence rate(A), prevalence rate(B), years lived with disability rate(C) of uterine fibroids in global and five-SDI quintiles from 1990 to 2019. Note: A-C use the same set of legends

**Fig. 5:**
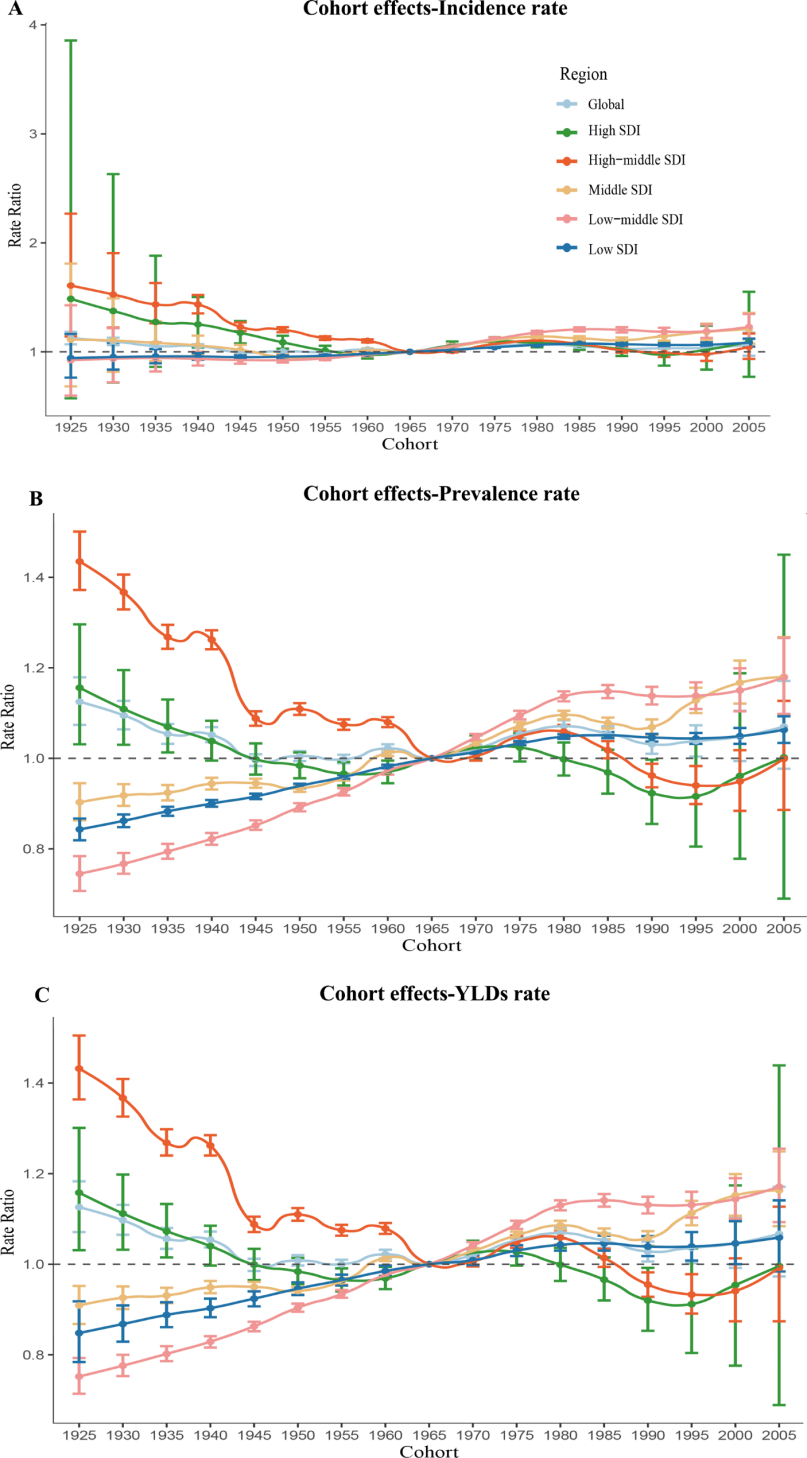
Parameter estimates of cohort effects on incidence rate(A), prevalence rate(B), years lived with disability rate(C) of uterine fibroids in global and five-SDI quintiles from 1990 to 2019. Note: A-C use the same set of legends

Figures 3, 4 and 5 exhibit the estimated effects of age, period and cohort on uterine fibroids incidence rate, prevalence rate and YLDs rate by APC model. Generally, similar patterns in age effects of incidence rate were found across all SDI regions. In the age group of 10–14 years to 35–39 years, the risk increased with advancing age and peaked at the age of 35–39 years. After age of 40 years, the risk sharply declined with age, with the lowest risk at the oldest age group. Likewise, the age effect of prevalence rate and YLDs rate on uterine fibroids was analogously in all SDI regions with increasing risk from 10 to 14 years (the lowest risk) to 40–44 years and the latter group having the highest risk. After the age of 45, the risk crisply declined with advancing age.

The effects of period effects in incidence rate, prevalence rate, and YLDs rate showed significantly differences across SDI regions over the study period. For high SDI and high-middle SDI countries, period effects presented a declining risk of incidence rate, prevalence rate and YLDs rate during 2005 to 2019 years. On the contrary, period effects showed an increasing risk of incidence rate, prevalence rate and YLDs rate across middle SDI regions, low-middle SDI regions and low SDI region over the past 15 years. Compared with 2000–2004, the RRs of incidence rate in 2015–2019 ranged from 0.88 (95% CI: 0.83 to 0.94) in high SDI countries to 1.10 (95% CI: 1.08 to 1.13) in low-middle SDI countries. Compared with 2000–2004, the RRs of prevalence rate in 2015–2019 ranged from 0.92 (95% CI: 0.89 to 0.94) in high-middle SDI to 1.13 (95% CI: 1.12 to 1.14) in low-middle SDI countries. In comparison to 2000–2004, the RRs of YLDs rate in 2015–2019 ranged from 0.91 (95% CI: 0.90 to 0.92) in high-middle SDI to 1.12 (95% CI: 1.12 to 1.13).

Like the effects of period, the cohort effects on incidence rate, prevalence rate, and YLDs rate across all SDI countries were found significantly differences. Middle SDI countries, low-middle SDI countries and low SDI countries had increasing risk of incidence rate, prevalence rate, and YLDs rate in those born after 1965, whereas the risk in high SDI countries and high-middle SDI countries had little change over different cohorts. Compared with individuals born in the cohort of 1965, the relative cohort risk of incidence rate for individual born in the 2005 cohort ranged from 1.05 (95% CI: 0.94 to 1.17) in high- middle SDI countries to 1.23 (95% CI: 1.12 to 1.35) in low-middle SDI countries. Compared with individuals born in the cohort of 1965, the relative cohort risk of prevalence rate for individual born in the 2005 cohort ranged from 1.00 (95% CI: 0.69 to 1.45) in high-middle SDI countries to 1.18 (95% CI: 1.10 to 1.27) in low-middle SDI countries. Compared with individuals born in the cohort of 1965, the relative cohort risk of YLDs rate for individual born in the 2005 cohort ranged from 0.99 (95% CI: 0.87 to 1.13) in high-middle SDI countries to 1.17 (95% CI: 1.09 to 1.26) in low-middle SDI countries.

## Discussion

High-SDI and high-middle SDI quintiles with decreasing trends, increasing trends were observed in middle SDI, low-middle SDI and low SDI quintiles in annual percentage change of incidence rate, prevalence rate and YLDs rate over the past 30 years. Across the 204 countries and territories, large majority of countries displayed an upward trend in incidence rate, prevalence rate, and YLDs rate. Judging from the age, the risk of uterine fibroids in different age group were unimodal distributed and peaked in the age group of 35 to 39 years in incidence rate and peaked in the age group of 40 to 44 years in prevalence rate and YLDs rate. Unfavorable period and cohort effects have been observed in middle SDI, low-middle SDI and low SDI quintiles over the recent 15 years and the birth cohort later than 1965.

Globally, in 2019 uterine fibroids accounted for 9.64 million incident cases, 226.05 million prevalent cases and 1.25 million the number of YLDs, a significant increase compared with the number in 1990. The annual percentage change in incidence rate, prevalence rate and YLDs rate of uterine fibroids increased in middle SDI, low-middle SDI and low SDI countries such as African countries, India and Brazil. 33 of the 47 least-developed countries in the world are located in African [27] where has been reported as having the largest population of black women who had 3–4 times greater rates of occurring uterine fibroids than the other races and 70–80% of black women will experience fibroids during their lifetime [1, 28]. Due to lack of knowledge, wrongful deep-seated cultural beliefs, poverty, without health insurance and transportation limitations, many African women with fibroids delay presenting for evaluation and management for an extended period of time [29, 30]. Oftentimes, African women present late in the course of disease when fibroids have grown to unmanageable proportions and are causing debilitating health issues, such as severe hemorrhage and grave anemia. Not only that, lacking highly skilled experts, poor quality of services, unaffordable pharmaceutical costs and poor nutritional status together aggravates morbidity and mortality in African countries [30, 31]. On this ground, education and increased awareness of the symptoms of uterine fibroids as well as international collaborations and support such as provide healthcare and transfer skills, medical supplies donation, facilities construction may ameliorate the current situation [30, 32].

As large population and economic rapid development countries, the increased trend of uterine fibroids in India and Brazil present profound effect worldwide. In the last two decades, with the rapidly developed of economic growth and progressing in the construction of primary health service system, a substantial improvement in reducing mortality of women was observed in both India and Brazil [33]. The possible reason for an increasing trend of annual percentage change in incidence rate, prevalence rate of uterine fibroids is that the diagnostic rate growing with the improvement of medical resources and medical equipment as well as the increasing of individual’s health awareness. Besides, driven by economic development and urbanization, the surge of animal-based protein consumption especially meat intake may be another factor that increased the risk of uterine fibroids [1, 34]. Despite the economic development and all the efforts of government, India and Brazil are the two countries with highly levels of income inequality and stark health inequalities. The poorest, the least educated and those residing in rural areas women had lower health care and worse health outcome [35–37]. Consequently, more resources should be put into medical field in order to expand the primary healthcare coverage, shrink the medical gap between urban and rural and improve the technical level of medical staff.

United Kingdom and New Zealand, both are high SDI countries, had low ASR for incidence, ASR for prevalence, ASR for YLDs of uterine fibroids and showed decreased trend of annual percentage change over the past 30 years. In general, national health insurance system with universal coverage, advanced medical facilities, higher educational attainment and awareness of seeking medical assistance makes uterine fibroids be diagnosed at early stages and effectively treated, so as to drastically decreased the complications of uterine fibroids. In addition, women in such countries are easily get access to quality hospitals with regulated standards of care and highly skilled experts, and patients are presented with an array of surgical options depending on the severity of patient’s tumor and burden of disease [11, 38, 39].

As is well known, age is a vital risk factor for uterine fibroids. Our study demonstrated that the risk of incidence rate increased significantly from 10 to 14 years to 35–39 years and then decreased with the advancing age. The risk of prevalence rate and YLDs rate increased from 10 to 14 years to 40–44 years and decreased with increasing age. Previous studies have shown that uterine fibroids tend to increase with age through the reproductive years and decline in postmenopausal years [16, 40–42] which were similar to the findings from our study. In fact, uterine fibroids are highly dependent on the ovarian steroids estrogen and progesterone [43]. Ovarian activity is essential for the growth of uterine fibroids which have not been detected in prepubertal girls and most of uterine fibroids shrink after menopause [3, 44]. Consequently, screening women in their reproductive years for uterine fibroids, long-term follow-up and appropriate treatment are necessary initiatives to mitigate the global health burden.

Period effects often reflect the impact of social, economic, and medical factors on disease across all age groups. The RRs of the period on uterine fibroids of incidence rate, prevalence rate and YLDs rate revealed that the risk of developing uterine fibroids decreased in high SDI and high-middle SDI countries from 2005 to 2019. In contrast, the RRs were increased in middle SDI, low-middle SDI and low SDI countries over the past 15 years. Yu O et al. performed a retrospective population-based cohort study of American women from 2005 to 2014 so as to seek the 10-year secular trend of uterine fibroids. Their research revealed that overall age-adjusted estimated incidence rate declined during the 10-year interval [42]. The decline in trend may be due, in part, to the decrease in percentage of hysterectomies in recent years that the proportion of cases diagnosed by pathology have been reduced [45, 46]. Another reason for the declining temporary trend could be the diminished use of menopausal hormone therapy over the past few years [42]. In middle SDI and low-middle SDI countries, the upward trend in uterine fibroids could be the result of improvement of economic and medical environment as well as the increase in health awareness that led to the increase of diagnostic rate [47, 48]. The RRs were increased in low SDI countries may be contributed to the poverty and lack of medical resources following wars and natural disasters [28, 30].

Cohort effects on uterine fibroids showed an increasing trend from 1965 cohort to 2005 cohort in Middle SDI, low-middle SDI and low SDI countries. This trend probably arose because with the acceleration of economic development, the dietary structure and lifestyle have changed greatly with a high intake of red meat, processed meats and low intake of vegetables and a low amount of exercise which are risk factors for uterine fibroids [49]. Besides, following the development of industry, the probability of exposure to plasticizers, dioxins, phthalates or other endocrine disrupting chemicals that contribute to the occurrence of uterine fibroids [50, 51].

Our study provides a more granular understanding of uterine fibroids trends through the finest use of data to attain public health insights. However, some limitations are associated with our study. First, this study uses data from a single source, derived entirely from GBD 2019 research results. Second, many countries, especially African countries, do not have primary data on uterine fibroids. The estimates data were mainly generated from predicted covariates and neighboring locations which might affect estimates of temporary trend in some low SDI countries. Thirdly, we appraised the age/period/cohort effects of uterine fibroids based on the estimated cross-sectional data of GBD from 1990 to 2019, which were not a cohort study. These limitations potentially cause sufficiently large differences between primary data and estimated data that could not represent the true burden of uterine fibroids in missing data countries especially the African countries that might affect the governments’ attention and decision-making on health expenditure which somewhat limit the efficient development of therapies for uterine fibroids. Besides, not accurate estimated data might lead to insufficient awareness of uterine fibroids for women in low SDI countries so as to delay the medical presentation, worsen the condition, lower the quality of life, and increase the mortality. In conclusion, more and better primary data collection is a fundamental improvement to address these limitations.

## Conclusion

Uterine fibroids are a major public health problem among women of reproductive age. Globally, incident cases, prevalent cases, the number of YLDs of uterine fibroids increased from 1990 to 2019. High SDI and high-middle SDI quintiles with decreasing trends of incidence rate, prevalence rate and YLDs rate, increasing trends were observed in middle SDI, low-middle SDI and low SDI quintiles over the past 30 years. Reproductive women aged 35–39 years had the highest risk of incidence rate and aged 40–44 years had the highest risk of prevalence rate and YLDs rate. Unfavorable period and cohort effects have been observed in middle SDI, low-middle SDI and low SDI quintiles over the recent years. The healthcare system needs to be strengthened to improve screening and management of uterine fibroids in most developing countries. Further efforts should also be improved international health assistance to low SDI countries.

## Electronic supplementary material

Below is the link to the electronic supplementary material.

Supplementary Material 1

## Data Availability

The datasets generated and/or analyzed during the current study are publicly available in the Global Health Data Exchange (GHDx) repository, http://ghdx.healthdata.org/gbd-results-tool, which is supported by the Institute for Health Metrics and Evaluation (IHME), University of Washington, USA.

## Abbreviations

APC: age-period-cohort
ASR: age-standardized rate
CI: confidence intervals
GBD 2019: Global Burden of Disease 2019 study
RRs: relative risks
SDI: Socio-demographic Index
UIs: uncertainty intervals
YLDs: years lived with disability
