# Supplementary material for: Global, regional, and national time trends in incidence, prevalence, years lived with disability for uterine fibroids, 1990–2019: an age-period-cohort analysis for the global burden of disease 2019 study
Source: BMC Public Health. 2023 May 19;23:916. doi: 10.1186/s12889-023-15765-x (PMC10199532; doi:10.1186/s12889-023-15765-x)
Supplement: Supplementary file 1 — Supplementary Material 1 [file 12889_2023_15765_MOESM1_ESM.docx]

**Supplementary materials to**

**Global, regional, and national time trends in incidence, prevalence, years lived with disability for uterine fibroids, 1990-2019: An age-period-cohort analysis for the Global Burden of Disease 2019 study**

| **Table S1.** The incidence and age-standardized rate of uterine fibroids in 1990 and 2019, and the temporal trends from 1990 to 2019 at national level. | | | | | |
| --- | --- | --- | --- | --- | --- |
| Incidence | | | | | |
| Nation | Cases in1990 | ASR in 1990  (per 100 000 persons) | Cases in 2019 | ASR in 2019  (per 100 000persons) | Net Drift  (%, per year) |
| Afghanistan | 4248  (3170-5593) | 102.13  (75.75-138.27) | 16547  (12375-22240) | 109.6  (81.91-147.81) | 0.24 (0.21-0.28) |
| Albania | 3753  (2775-4900) | 254.57  (188.28-333.46) | 3477  (2582-4596) | 268.7  (198.46-354.22) | 0.19  (0.17-0.2) |
| Algeria | 12331  (9076-16760) | 118.91  (87.26-159.79) | 30048  (21940-40939) | 125.77  (92.69-170.6) | 0.19  (0.17-0.21) |
| American Samoa | 30  (22-41) | 132.9  (96.45-178.49) | 36  (27-47) | 142.6  (105.67-189.48) | 0.24  (0.23-0.26) |
| Andorra | 104  (75-141) | 326.1  (235.34-434.89) | 159  (114-214) | 337.33  (245.2-447.24) | 0.12  (0.09-0.15) |
| Angola | 9739  (7187-13098) | 232.56  (171.97-310.92) | 33342  (24577-44881) | 257.14  (188.6-343.7) | 0.35  (0.34-0.36) |
| Antigua and Barbuda | 110  (81-150) | 345.16  (253.52-465.88) | 183  (134-246) | 358.16  (263.89-479.15) | 0.13  (0.11-0.14) |
| Argentina | 39099  (28212-51991) | 245.05  (176.64-326.22) | 63099  (45264-85093) | 260.27  (186.77-351.49) | 0.21  (0.2-0.22) |
| Armenia | 6836  (4990-9329) | 400.93  (293.41-534.46) | 7258  (5236-9777) | 425.26  (308.06-568.24) | 0.21  (0.19-0.22) |
| Australia | 7754  (5604-10356) | 83.23  (60.63-110.95) | 11033  (8002-14614) | 86.13  (62.44-114.45) | 0.11  (0.09-0.14) |
| Austria | 14936  (11187-19247) | 366.91  (276.19-474.5) | 14938  (10983-19649) | 336.74  (247.73-443.45) | -0.30  (-0.32--0.28) |
| Azerbaijan | 13132  (9592-17528) | 391.81  (287.48-513.02) | 25163  (18173-34021) | 423.79  (308.53-565.63) | 0.27  (0.24-0.29) |
| Bahamas | 498  (358-676) | 357.27  (255.86-476.68) | 785  (566-1056) | 364.05  (262.32-488.36) | 0.06  (0.06-0.07) |
| Bahrain | 292  (211-406) | 123.21  (92.26-166.7) | 910  (658-1243) | 124.84  (92.62-169.47) | 0.05  (0.04-0.05) |
| Bangladesh | 71692  (52739-97485) | 169.64  (125.27-226.81) | 164952  (119961-224242) | 188.86  (137.11-257.58) | 0.37  (0.34-0.39) |
| Barbados | 503  (361-688) | 357.32  (258.84-484.4) | 547  (405-725) | 363.29  (267.83-487.8) | 0.05  (0.05-0.06) |
| Belarus | 29605  (21508-39276) | 544.4  (399.4-717.35) | 29263  (21365-38475) | 561.55  (414.38-740.88) | 0.11%  (0.1-0.12) |
| Belgium | 17281  (12560-23151) | 330.59  (241.81-441.46) | 18445  (13494-24837) | 336.65  (245.85-448.81) | 0.06  (0.04-0.08) |
| Belize | 253  (185-349) | 348.3  (252.35-470.65) | 793  (581-1045) | 365.55  (266.58-481.8) | 0.16  (0.15-0.18) |
| Benin | 4719  (3487-6451) | 235.19  (174.33-314.08) | 13879  (10273-18487) | 256.47  (188.99-338.79) | 0.29  (0.26-0.32) |
| Bermuda | 142  (101-191) | 373.48  (272.86-492.85) | 118  (85-159) | 376.32  (272.95-515.87) | 0.03  (0.02-0.04) |
| Bhutan | 421  (309-563) | 176.8  (129.72-235.9) | 810  (589-1085) | 199.64  (145.92-267.08) | 0.42  (0.39-0.45) |
| Bolivia (Plurinational State of) | 13933  (10171-18696) | 491.1  (355.18-660.66) | 30369  (22070-41033) | 504.7  (367.49-683.03) | 0.10  (0.09-0.11) |
| Bosnia and Herzegovina | 5842  (4324-7759) | 247.9  (184.65-329.9) | 4649  (3365-6166) | 271.73  (196.98-356.83) | 0.32  (0.29-0.35) |
| Botswana | 2510  (1852-3382) | 437.56  (322.53-586.08) | 6532  (4689-8848) | 470.32  (343.39-630.07) | 0.29  (0.23-0.35) |
| Brazil | 132106  (99811-171427) | 178.52  (135.43-230.6) | 343686  (254994-449989) | 274.44  (203.73-358.2) | 1.47  (1.38-1.56) |
| Brunei Darussalam | 293  (210-401) | 201.25  (147.2-270.71) | 563  (411-758) | 216.06  (159.57-290.12) | 0.23  (0.19-0.28) |
| Bulgaria | 12363  (8930-16648) | 269.15  (196.73-361.33) | 10043  (7322-13606) | 281.18  (205.62-371.24) | 0.15  (0.14-0.16) |
| Burkina Faso | 8735  (6499-11742) | 228.32  (168.07-303.93) | 24538  (18103-32814) | 246.73  (182.78-329.09) | 0.27  (0.26-0.28) |
| Burundi | 4140  (3044-5629) | 179.5  (131.72-243.81) | 8936  (6590-11975) | 179.48  (132.86-240.23) | 0  (-0.02-0.02) |
| Cabo Verde | 330  (244-452) | 235.85  (174.71-315.97) | 765  (566-1028) | 256.69  (189.58-340.45) | 0.28  (0.24-0.33) |
| Cambodia | 5980  (4421-8142) | 126.13  (93.08-169.69) | 12389  (9141-16933) | 138.19  (102.38-187.32) | 0.32  (0.3-0.34) |
| Cameroon | 10754  (7923-14348) | 253.12  (184.85-335.13) | 35260  (25690-47751) | 262.61  (191.75-352.35) | 0.12  (0.07-0.16) |
| Canada | 19569  (14042-26079) | 121.22  (87.93-160.73) | 21616  (15726-29221) | 121.64  (88.37-165.3) | 0.01  (-0.04-0.05) |
| Central African Republic | 2808  (2051-3839) | 241.58  (177.78-324.34) | 5801  (4277-7774) | 243.71  (179.42-325.1) | 0.03  (0.02-0.05) |
| Chad | 5057  (3760-6780) | 211.26  (156.74-281.85) | 14245  (10666-19014) | 230.54  (171.51-310.72) | 0.30  (0.27-0.33) |
| Chile | 16973  (12373-22922) | 242.34  (177.44-327.14) | 25525  (18279-34876) | 262.97  (187.61-359.79) | 0.27  (0.22-0.33) |
| China | 747648  (547009-1012871) | 120.98  (88.37-163.32) | 1046738  (786589-1395048) | 133.8  (100.74-177.06) | 0.35  (0.31-0.4) |
| Colombia | 64163  (45742-86336) | 406.17  (292.46-549.22) | 104594  (75681-139032) | 408.2  (294.79-542.27) | 0.03  (-0.02-0.08) |
| Comoros | 356  (265-478) | 193.71  (143.42-260.02) | 689  (510-905) | 195.35  (144.78-257.76) | 0.03  (0.02-0.04) |
| Congo | 2426  (1801-3273) | 245.8  (180.89-330.5) | 6890  (5025-9291) | 258.82  (188.65-345.03) | 0.18  (0.17-0.19) |
| Cook Islands | 11  (8-15) | 133.18  (98.39-178.48) | 12  (9-17) | 144.83  (107.24-194.89) | 0.29  (0.28-0.3) |
| Costa Rica | 6006  (4277-7963) | 412.27  (293.65-549.56) | 11459  (8146-15483) | 421.32  (300.54-567.94) | 0.07  (0.05-0.08) |
| Croatia | 7175  (5172-9539) | 274.47  (199.35-360.3) | 6064  (4418-8028) | 287.96  (211.4-373.55) | 0.19  (0.11-0.26) |
| Cuba | 20946  (15402-28162) | 366.99  (267.95-487.57) | 20346  (14770-27291) | 378.48  (274.36-510.42) | 0.11  (0.09-0.13) |
| Cyprus | 1235  (898-1665) | 302.08  (219.94-406.99) | 2653  (1881-3588) | 321.59  (231.99-433.71) | 0.21  (0.14-0.27) |
| Czechia | 17775  (12677-24225) | 307.52  (224.8-411.73) | 18429  (13425-25087) | 320.27  (234.88-430.5) | 0.14  (0.08-0.2) |
| Côte d'Ivoire | 11477  (8477-15730) | 237.36  (175.14-319.94) | 31362  (22733-42802) | 259.03  (189.12-347.22) | 0.29  (0.25-0.33) |
| Democratic People's Republic of Korea | 10314  (7615-13882) | 98.63  (72.53-131.78) | 14035  (10328-18701) | 103.72  (75.68-138.13) | 0.18  (0.16-0.2) |
| Democratic Republic of the Congo | 35710  (26213-47626) | 232.51  (170.55-308.29) | 90677  (66569-121900) | 244.38  (178.26-332.06) | 0.17  (0.13-0.21) |
| Denmark | 7616  (5505-10231) | 275.28  (199.08-370.45) | 7367  (5406-9823) | 275.92  (201.07-372.8) | 0.01  (-0.01-0.03) |
| Djibouti | 344  (257-456) | 186.89  (138.99-245.23) | 1312  (942-1818) | 203.21  (148.61-276.49) | 0.29  (0.28-0.3) |
| Dominica | 105  (78-141) | 345.38  (251.18-459.45) | 119  (87-159) | 356.29  (260.39-476.14) | 0.11  (0.1-0.11) |
| Dominican Republic | 11565  (8452-15711) | 342.5  (248.6-464.31) | 20615  (15098-27490) | 366  (267.17-491) | 0.23  (0.22-0.25) |
| Ecuador | 23601  (17589-31570) | 510.95  (378.31-678.39) | 46289  (35342-59347) | 509.85  (388.67-652.39) | -0.01  (-0.03-0.02) |
| Egypt | 30198  (22439-40775) | 122.74  (90.83-165.71) | 63200  (46470-84651) | 127.17  (94.09-169.65) | 0.12  (0.11-0.13) |
| El Salvador | 8770  (6262-11702) | 394.71  (280.93-526.14) | 13787  (9925-18522) | 407.61  (293.25-543.05) | 0.11  (0.09-0.14) |
| Equatorial Guinea | 420  (310-559) | 232.8  (171.68-307.38) | 1652  (1208-2208) | 269.44  (197.21-355.67) | 0.51  (0.47-0.55) |
| Eritrea | 2128  (1574-2857) | 173.8  (128.1-234.12) | 5662  (4199-7554) | 184.44  (136.2-246.96) | 0.20  (0.19-0.21) |
| Estonia | 4609  (3341-6032) | 553.02  (405.36-718.49) | 3749  (2721-4995) | 572.48  (419.66-766.16) | 0.12  (0.07-0.17) |
| Eswatini | 1553  (1140-2103) | 453.65  (334.77-605.76) | 2798  (2047-3826) | 461.99  (335.56-623.4) | 0.07  (0.03-0.11) |
| Ethiopia | 37203  (27741-49351) | 180.98  (135.54-239.32) | 90764  (67131-120938) | 193.82  (143.86-259.3) | 0.24  (0.23-0.26) |
| Fiji | 487  (359-654) | 129.22  (94.9-172.34) | 651  (474-875) | 140.54  (102.81-188.53) | 0.29  (0.27-0.31) |
| Finland | 8647  (6260-11482) | 305.21  (220.74-405.24) | 7783  (5700-10489) | 310.07  (227.04-416.77) | 0.04  (-0.02-0.09) |
| France | 116661  (84272-157495) | 379.71  (276.19-510.52) | 121147  (87197-161723) | 392.94  (280.97-526.98) | 0.14  (0.09-0.18) |
| Gabon | 972  (715-1319) | 251.29  (183.32-337.59) | 2490  (1827-3310) | 270.12  (197.98-357.66) | 0.26  (0.22-0.29) |
| Gambia | 968  (707-1320) | 240.1  (176.45-319.7) | 2654  (1955-3608) | 256.13  (188.65-342.77) | 0.22  (0.16-0.27) |
| Georgia | 11367  (8324-15218) | 404.25  (292.49-541.89) | 9024  (6734-11657) | 480.51  (361.3-620.03) | 0.59  (0.55-0.63) |
| Germany | 166212  (120878-223957) | 398.38  (289.78-537.99) | 159137  (114679-219860) | 409.68  (295.67-563.31) | 0.10  (0.08-0.13) |
| Ghana | 15019  (10966-20293) | 232.73  (171.81-312.32) | 40860  (29983-55262) | 246.31  (181.77-328.64) | 0.19  (0.17-0.21) |
| Greece | 17476  (12834-23754) | 335.22  (246.49-457.28) | 17914  (13045-24233) | 345.79  (253.36-461.54) | 0.11  (0.09-0.12) |
| Greenland | 47  (34-65) | 157.06  (113.07-218.4) | 44  (31-59) | 162.12  (115.2-216.81) | 0.11  (0.1-0.12) |
| Grenada | 121  (88-165) | 336.82  (244.95-446.32) | 179  (132-239) | 353.56  (258.16-474.84) | 0.17  (0.14-0.21) |
| Guam | 94  (69-131) | 132.87  (98.66-181.72) | 110  (82-148) | 144.6  (105.84-197) | 0.29  (0.27-0.31) |
| Guatemala | 12038  (8791-15926) | 382.14  (276.85-509.78) | 37099  (26541-50070) | 396.5  (283.61-537.12) | 0.12  (0.08-0.15) |
| Guinea | 6039  (4425-8063) | 227.37  (166.46-302.19) | 13652  (10031-18622) | 240.37  (177.37-323.95) | 0.19  (0.17-0.21) |
| Guinea-Bissau | 993  (731-1355) | 231.42  (170.91-311.01) | 2224  (1633-2985) | 240.49  (177.51-316.38) | 0.12%  (0.07-0.17) |
| Guyana | 1258  (919-1694) | 339.61  (247.3-453.99) | 1414  (1034-1860) | 356.62  (259.69-474.22) | 0.17  (0.16-0.18) |
| Haiti | 9078  (6721-12367) | 316.74  (233.56-419.84) | 22223  (16236-29997) | 322.62  (237.05-431.8) | 0.06  (0.05-0.07) |
| Honduras | 7004  (5106-9336) | 388.1  (281.27-523.38) | 20248  (14446-27177) | 407.06  (289.16-544.67) | 0.17  (0.13-0.2) |
| Hungary | 15420  (11175-20459) | 269.2  (196.29-354.65) | 14551  (10426-20002) | 275.99  (202.45-366.15) | 0.09  (0.08-0.1) |
| Iceland | 410  (294-536) | 311.87  (224.23-407.48) | 517  (379-696) | 307.71  (225.02-414.32) | -0.07  (-0.16-0.03) |
| India | 936198  (687559-1240331) | 247.53  (183.37-329.09) | 2321569  (1703412-3089768) | 319.78  (235.53-425.12) | 0.89  (0.85-0.93) |
| Indonesia | 104029  (77451-140540) | 113.88  (85.22-151.97) | 159243  (120167-209852) | 111.62  (84.52-148.36) | -0.06  (-0.09--0.03) |
| Iran (Islamic Republic of) | 32080  (23753-43585) | 135.66  (101.18-182.13) | 71235  (52208-95802) | 137.75  (103.06-183.65) | 0.01  (-0.09-0.11) |
| Iraq | 8400  (6207-11496) | 123.11  (91.33-167.49) | 26096  (19352-34996) | 122.92  (91.13-163.74) | -0.01  (-0.03-0.02) |
| Ireland | 5749  (4189-7707) | 327.99  (240.59-439.39) | 8874  (6350-11854) | 333.82  (241.25-443.82) | 0.06  (0.05-0.08) |
| Israel | 7844  (5599-10658) | 320.14  (231.52-434.88) | 14666  (10646-19204) | 328.23  (237.11-428.25) | 0.09  (0.08-0.1) |
| Italy | 86578  (64074-115752) | 294.46  (216.99-393.28) | 74881  (57271-97064) | 282.66  (214.56-369.65) | -0.14  (-0.17--0.11) |
| Jamaica | 3747  (2766-5150) | 348.59  (257.62-464.26) | 5665  (4093-7573) | 365.22  (263.77-487.23) | 0.16  (0.15-0.17) |
| Japan | 197715  (146501-262256) | 327.97  (239.45-442.2) | 168216  (127141-219698) | 354.24  (266.99-465.31) | 0.26  (0.24-0.29) |
| Jordan | 1704  (1275-2317) | 121.1  (90.21-163.69) | 6899  (5141-9399) | 125.65  (94.06-170.19) | 0.13  (0.11-0.14) |
| Kazakhstan | 32886  (23945-44550) | 403.73  (295.04-536.47) | 43811  (31911-58514) | 430.22  (315.69-574.04) | 0.22  (0.2-0.23) |
| Kenya | 17791  (13263-23807) | 202.44  (150.84-267.1) | 49993  (37202-66448) | 207.81  (154.88-274.09) | 0.09  (0.08-0.09) |
| Kiribati | 45  (33-61) | 125.11  (92.96-168.8) | 85  (62-116) | 138.37  (101.54-187.58) | 0.35  (0.32-0.37) |
| Kuwait | 1085  (786-1493) | 126.35  (93.99-167.53) | 4221  (3005-5798) | 130.57  (96.4-175.13) | 0.12  (0.09-0.15) |
| Kyrgyzstan | 7301  (5397-9934) | 384.83  (281.43-513.27) | 13245  (9695-17337) | 402.44  (294.53-524.96) | 0.15  (0.11-0.18) |
| Lao People's Democratic Republic | 2318  (1708-3127) | 129  (94.11-174.67) | 5442  (4035-7363) | 142.83  (105.42-190.34) | 0.35  (0.34-0.37) |
| Latvia | 8850  (6519-11824) | 641.77  (471.65-860.51) | 6265  (4592-8293) | 667.14  (492.3-884.54) | 0.13  (0.09-0.17) |
| Lebanon | 1811  (1348-2420) | 123.77  (91.66-165.54) | 3873  (2844-5220) | 129.27  (95.83-173.26) | 0.15  (0.14-0.16) |
| Lesotho | 3252  (2386-4374) | 427.96  (311.18-571.01) | 5021  (3687-6740) | 459.6  (337.68-613.03) | 0.25  (0.22-0.28) |
| Liberia | 1941  (1429-2617) | 238.39  (175.5-320.5) | 5771  (4256-7693) | 251.27  (185.63-331.98) | 0.18  (0.16-0.2) |
| Libya | 1843  (1379-2473) | 122.1  (90.03-164.5) | 5016  (3705-6783) | 122.64  (91.24-165.98) | 0.01  (0-0.03) |
| Lithuania | 8623  (6304-11381) | 451.97  (329.31-598.89) | 6220  (4562-8207) | 470.92  (347.14-620.54) | 0.13  (0.1-0.16) |
| Luxembourg | 888  (629-1190) | 415.91  (297.08-554.75) | 1449  (1056-1992) | 422.9  (310.32-576.34) | 0.05  (-0.02-0.12) |
| Madagascar | 8834  (6585-11782) | 185.47  (137.92-246.99) | 22376  (16693-29989) | 185.03  (137.94-246.13) | -0.01  (-0.03-0.01) |
| Malawi | 7519  (5602-10083) | 194.56  (143.74-255.22) | 15817  (11566-21404) | 193.9  (142.22-264.14) | -0.01  (-0.03-0.01) |
| Malaysia | 12563  (9332-17060) | 143.22  (106.01-192.08) | 26521  (19551-35554) | 157.07  (116.35-210.45) | 0.32  (0.31-0.33) |
| Maldives | 109  (81-149) | 130.71  (96.63-176.21) | 379  (274-520) | 152.07  (111.81-205.8) | 0.52  (0.51-0.54) |
| Mali | 7612  (5727-10203) | 216.22  (161.58-291.94) | 20781  (15257-28086) | 238.51  (174.28-317.88) | 0.33  (0.3-0.36) |
| Malta | 616  (442-834) | 293.92  (211.23-397.22) | 664  (481-889) | 304.23  (220.94-405.47) | 0.11  (0.07-0.14) |
| Marshall Islands | 21  (16-29) | 119.43  (88.02-161.79) | 39  (29-53) | 132.22  (97.68-179.22) | 0.35  (0.34-0.37) |
| Mauritania | 2066  (1523-2785) | 242.28  (178.92-327.14) | 4720  (3479-6273) | 259.15  (189.94-343.91) | 0.23  (0.21-0.24) |
| Mauritius | 867  (637-1170) | 141.97  (106.01-188.79) | 1022  (745-1353) | 153.61  (112.31-205) | 0.27  (0.24-0.3) |
| Mexico | 179576  (130351-238015) | 471.91  (343.41-627.25) | 315166  (233205-414672) | 458.89  (340.19-603.63) | -0.10  (-0.11--0.09) |
| Micronesia (Federated States of) | 53  (40-73) | 125.62  (93.72-171.04) | 66  (49-89) | 135.5  (99.71-184.27) | 0.26  (0.25-0.27) |
| Monaco | 53  (39-72) | 335.27  (245.27-448.64) | 56  (40-75) | 339.87  (245.41-456.32) | 0.05  (0.03-0.07) |
| Mongolia | 3260  (2406-4327) | 390.29  (288.64-517.27) | 8244  (6010-11088) | 414.78  (305.78-555.17) | 0.21  (0.17-0.24) |
| Montenegro | 849  (613-1126) | 268.11  (194.21-357.5) | 924  (667-1248) | 282.36  (205.85-379.81) | 0.18  (0.15-0.21) |
| Morocco | 13747  (10214-18457) | 117.66  (87.14-157.68) | 23765  (17710-32266) | 121.45  (90.56-165.04) | 0.11  (0.1-0.12) |
| Mozambique | 10220  (7590-13676) | 182.01  (135.32-245.49) | 24168  (18035-32726) | 195.74  (144.75-264.87) | 0.26  (0.24-0.27) |
| Myanmar | 25024  (18312-34064) | 128.69  (95.38-174.01) | 43408  (31890-57301) | 144.03  (105.74-190.34) | 0.39  (0.37-0.41) |
| Namibia | 2607  (1911-3470) | 435.98  (317.69-579.5) | 5588  (4087-7489) | 448.86  (329.81-603.95) | 0.10  (0.08-0.13) |
| Nauru | 6  (5-8) | 129.51  (95.79-171.99) | 8  (6-10) | 140.21  (104.15-187.86) | 0.28  (0.26-0.29) |
| Nepal | 10787  (7995-14570) | 130.81  (96.58-174.27) | 22302  (16737-28990) | 135.16  (101.42-176.5) | 0.10  (0.05-0.15) |
| Netherlands | 27295  (19633-36539) | 327.13  (235.99-439.28) | 26164  (19026-35377) | 330.46  (237.72-448.95) | 0.04  (0.03-0.05) |
| New Zealand | 1666  (1224-2269) | 90.96  (67.03-123.73) | 1818  (1382-2329) | 81.98  (62.13-104.51) | -0.37  (-0.42--0.33) |
| Nicaragua | 5902  (4230-7939) | 392.36  (281.67-531.93) | 14113  (10120-18947) | 407.61  (293.25-543.05) | 0.13  (0.1-0.16) |
| Niger | 6643  (4895-8858) | 216.32  (158.76-292.63) | 18794  (13883-25161) | 229.98  (167.33-307.9) | 0.21  (0.2-0.22) |
| Nigeria | 87960  (66063-118306) | 251.41  (187.43-331.94) | 248294  (186555-330627) | 261.25  (194.75-344.52) | 0.13  (0.09-0.16) |
| Niue | 1  (1-2) | 130.83  (96.25-176.13) | 1  (1-1) | 143.06  (104.13-192.69) | 0.31  (0.28-0.34) |
| North Macedonia | 2685  (1988-3559) | 256.87  (189.84-341.89) | 3188  (2326-4285) | 271.99  (200.44-361.1) | 0.20  (0.19-0.21) |
| Northern Mariana Islands | 37  (27-51) | 132.21  (96.67-176.51) | 24  (18-31) | 139.63  (103.72-186.41) | 0.19  (0.18-0.2) |
| Norway | 5044  (3662-6854) | 229.94  (168.11-312.03) | 6083  (4475-8274) | 231.69  (169.51-313.39) | 0  (-0.08-0.08) |
| Oman | 749  (545-1026) | 115.77  (85.66-154.99) | 2626  (1894-3606) | 125.38  (93.35-167.76) | 0.28  (0.26-0.3) |
| Pakistan | 83098  (61435-111014) | 201  (147.15-269.28) | 208014  (154879-275442) | 207.97  (155.45-278.02) | 0.12  (0.07-0.16) |
| Palau | 11  (8-14) | 132.22  (97.24-178.59) | 11  (8-15) | 143.25  (105.11-192.85) | 0.27  (0.25-0.3) |
| Palestine | 893  (662-1228) | 117.05  (86.04-158.97) | 2796  (2072-3802) | 120.47  (89.15-161.55) | 0.10  (0.08-0.11) |
| Panama | 4262  (3026-5694) | 386.64  (273.15-518.34) | 8436  (6049-11242) | 407.92  (292.34-544.3) | 0.19%  (0.16-0.22) |
| Papua New Guinea | 2121  (1552-2934) | 119  (87.25-162.18) | 6333  (4657-8566) | 129.74  (95.62-174.77) | 0.30  (0.29-0.32) |
| Paraguay | 3633  (2662-4874) | 208.22  (152.26-274.5) | 8022  (5813-10706) | 220.91  (160.2-293.88) | 0.21  (0.19-0.22) |
| Peru | 48096  (35003-65651) | 484.7  (349.1-652.22) | 93049  (67874-124620) | 507.69  (370.07-679.92) | 0.17  (0.15-0.19) |
| Philippines | 51967  (38861-69557) | 178.08  (132.57-237.64) | 112207  (84002-149388) | 201.51  (150.26-268.4) | 0.42  (0.4-0.45) |
| Poland | 43803  (31645-59194) | 210.99  (155.06-283.1) | 37972  (29303-48229) | 177.35  (139.82-222.67) | -0.59  (-0.67--0.51) |
| Portugal | 13441  (9798-17937) | 261.27  (190.01-348.7) | 13406  (9708-17715) | 243.4  (177.87-316.07) | -0.24  (-0.27--0.21) |
| Puerto Rico | 7009  (5104-9304) | 365.61  (265.54-488.06) | 6411  (4657-8560) | 377.64  (275.92-507.53) | 0.11  (0.1-0.13) |
| Qatar | 213  (153-298) | 124.09  (92.58-167.7) | 1396  (987-1931) | 127.37  (93.86-171.21) | 0.09  (0.06-0.11) |
| Republic of Korea | 40017  (29563-54586) | 151.89  (112.4-203.66) | 40268  (29589-54169) | 164.1  (120.22-222.3) | 0.25  (0.19-0.3) |
| Republic of Moldova | 12598  (9145-16924) | 520.58  (380.36-689.6) | 11695  (8490-15486) | 548.57  (401.82-720.89) | 0.18  (0.15-0.2) |
| Romania | 28287  (20731-37403) | 240.41  (175.97-317.4) | 25548  (18647-33788) | 253.56  (185.56-328.93) | 0.20  (0.15-0.25) |
| Russian Federation | 490160  (359564-654252) | 593.47  (440.26-785.78) | 480960  (353116-642742) | 586.64  (434.96-771.37) | -0.04  (-0.07--0.01) |
| Rwanda | 5574  (4074-7615) | 191.38  (140.61-257.24) | 12255  (8986-16584) | 197.82  (145.43-267.46) | 0.11  (0.1-0.13) |
| Saint Kitts and Nevis | 68  (49-94) | 356.14  (259-475.5) | 120  (88-160) | 368.42  (270.12-491.77) | 0.12  (0.11-0.13) |
| Saint Lucia | 209  (152-287) | 344.77  (247.66-468.11) | 344  (250-452) | 357.08  (259.47-471.97) | 0.12%  (0.1-0.14) |
| Saint Vincent and the Grenadines | 157  (115-214) | 337.93  (246.49-454.6) | 203  (147-269) | 355.64  (257.19-471.32) | 0.17  (0.16-0.19) |
| Samoa | 87  (64-117) | 133.65  (97.87-180.3) | 134  (99-181) | 144.25  (106.13-194.79) | 0.26  (0.25-0.27) |
| San Marino | 38  (28-51) | 331.9  (241.04-444.95) | 58  (42-80) | 336.44  (243.67-456.65) | 0.05  (0.03-0.07) |
| Sao Tome and Principe | 103  (76-139) | 232.99  (171.04-310.1) | 245  (181-327) | 247.4  (181.73-331.03) | 0.20  (0.15-0.25) |
| Saudi Arabia | 6919  (5181-9420) | 116.6  (86.93-158.03) | 26765  (19345-36639) | 125.36  (92.63-168.64) | 0.25  (0.23-0.26) |
| Senegal | 7355  (5479-9844) | 242.58  (178.97-326.76) | 16893  (12487-22544) | 247.42  (182.47-329.65) | 0.06  (0.05-0.08) |
| Serbia | 12310  (9049-16348) | 254.86  (186.48-337.54) | 12029  (8843-16084) | 272.57  (202.14-360.41) | 0.24  (0.22-0.26) |
| Seychelles | 49  (36-66) | 142.05  (105.6-190.46) | 77  (57-103) | 152.67  (113.18-203.63) | 0.25  (0.24-0.26) |
| Sierra Leone | 3822  (2787-5175) | 236.8  (174.12-314.4) | 9843  (7244-13231) | 256.26  (188.15-341.13) | 0.27  (0.25-0.29) |
| Singapore | 3852  (2835-5292) | 193.38  (143.5-264.52) | 6946  (5078-9442) | 209.51  (154.37-280.78) | 0.27  (0.26-0.29) |
| Slovakia | 8966  (6438-12042) | 314.52  (228.69-418.84) | 10130  (7296-13627) | 328.94  (240.39-439.77) | 0.15  (0.08-0.23) |
| Slovenia | 2705  (1996-3585) | 257.08  (191.06-341.06) | 2853  (2068-3816) | 272.69  (200.22-362.59) | 0.22  (0.16-0.28) |
| Solomon Islands | 156  (117-210) | 120.58  (89.41-161.38) | 415  (306-560) | 132.56  (98.26-178.2) | 0.33  (0.31-0.35) |
| Somalia | 5315  (3911-7178) | 183.19  (135.7-245.91) | 14821  (10966-19842) | 185.5  (136.91-247.49) | 0.04  (0.02-0.06) |
| South Africa | 94012  (70026-126027) | 513.65  (382.07-679.45) | 164170  (120325-220005) | 516.14  (384.71-683.55) | 0.02  (0-0.03) |
| South Sudan | 4068  (3048-5474) | 183.86  (136.03-248.01) | 7338  (5438-9879) | 186.16  (136.95-253.99) | 0.04  (0.03-0.05) |
| Spain | 78121  (56879-104109) | 408.26  (295.41-546.88) | 100266  (72378-137466) | 419.68  (307.17-570.64) | 0.11  (0.08-0.14) |
| Sri Lanka | 12215  (9101-16682) | 134.97  (100.44-182.2) | 16789  (12380-22488) | 145.84  (107.86-195.66) | 0.26  (0.25-0.28) |
| Sudan | 8550  (6337-11711) | 101.02  (75.49-137.72) | 22437  (16280-30352) | 111.69  (82.05-151.48) | 0.30  (0.33-0.36) |
| Suriname | 619  (456-831) | 351.91  (255.14-472.05) | 1095  (798-1456) | 370.33  (269.42-493.82) | 0.17  (0.16-0.19) |
| Sweden | 10999  (8008-14562) | 252.2  (184.21-335.21) | 11639  (8677-15539) | 240.93  (179.61-319.1) | -0.16  (-0.25--0.07) |
| Switzerland | 12461  (9000-16778) | 327.45  (235.96-440.72) | 14719  (10629-19647) | 329.57  (239.05-440.63) | 0.02  (0-0.04) |
| Syrian Arab Republic | 5727  (4223-7712) | 119.15  (88.29-159.59) | 8767  (6475-11650) | 122.54  (91.23-165.45) | 0.09  (0.08-0.11) |
| Taiwan (Province of China) | 12668  (9029-17148) | 109.59  (78.98-146.51) | 15484  (11207-20630) | 121.94  (88.97-164.32) | 0.37  (0.35-0.38) |
| Tajikistan | 7600  (5517-10211) | 383.49  (275.95-515.41) | 18111  (13210-24009) | 390.71  (283.84-521.7) | 0.06  (0.04-0.08) |
| Thailand | 43202  (32165-58265) | 137.59  (102.42-182.54) | 57535  (42233-76252) | 151.76  (111.79-203.08) | 0.33  (0.3-0.37) |
| Timor-Leste | 455  (340-618) | 126.47  (93.57-168.02) | 799  (599-1078) | 138.27  (103.58-186.84) | 0.31  (0.3-0.32) |
| Togo | 3523  (2587-4815) | 230.45  (168.52-313.22) | 9723  (7089-13187) | 244.75  (179.57-328.29) | 0.20  (0.18-0.23) |
| Tokelau | 1  (1-1) | 126.78  (93.72-170.51) | 1  (1-1) | 140.9  (104.52-187.91) | 0.36  (0.36-0.37) |
| Tonga | 52  (39-71) | 133.26  (98.27-178.48) | 68  (51-92) | 142.65  (105.51-192.82) | 0.24  (0.22-0.25) |
| Trinidad and Tobago | 2138  (1555-2897) | 352.46  (256.68-471.9) | 2796  (2008-3787) | 366.27  (265.69-494.43) | 0.13  (0.12-0.15) |
| Tunisia | 4573  (3419-6272) | 119.92  (89.45-161.72) | 8426  (6195-11355) | 126.77  (93.46-170.6) | 0.19  (0.14-0.23) |
| Turkey | 30620  (22621-41477) | 111.03  (82.7-149.54) | 55184  (40816-74399) | 118.94  (88.29-160.46) | 0.24  (0.22-0.26) |
| Turkmenistan | 5992  (4370-8078) | 394.44  (289.05-526.87) | 10536  (7626-14240) | 417.11  (302.61-562.64) | 0.18  (0.14-0.23) |
| Tuvalu | 6  (5-8) | 125.3  (92.74-167.02) | 7  (6-10) | 137.73  (102.85-184.91) | 0.33  (0.31-0.35) |
| Uganda | 12001  (8885-16055) | 185.03  (135.82-245.68) | 32125  (23913-43446) | 189.69  (140.56-255.81) | 0.09  (0.07-0.1) |
| Ukraine | 159155  (117310-210888) | 577.25  (426.21-757.97) | 144401  (105774-192149) | 578.21  (427.17-766.78) | 0  (0-0.01) |
| United Arab Emirates | 857  (609-1195) | 118.67  (87.66-159.43) | 5148  (3473-7334) | 125.59  (93.32-168.8) | 0.20  (0.19-0.2) |
| United Kingdom | 84178  (61878-113144) | 282.31  (206.75-376.8) | 86442  (63621-114921) | 255.12  (188.55-335.85) | -0.33  (-0.4--0.26) |
| United Republic of Tanzania | 20053  (14716-27165) | 193.49  (141.16-261.63) | 50656  (37710-67464) | 199.85  (147.6-264.45) | 0.11  (0.1-0.12) |
| United States of America | 302159  (226110-394024) | 210.73  (160.42-272.59) | 401245  (299246-516343) | 254.52  (189.81-328) | 0.65  (0.58-0.73) |
| United States Virgin Islands | 209  (151-282) | 356.18  (258.03-478.36) | 178  (129-240) | 369.82  (266.77-497.71) | 0.13  (0.12-0.14) |
| Uruguay | 3696  (2691-4902) | 243.57  (176.95-323.45) | 4555  (3312-6167) | 261.94  (189.81-357.54) | 0.25  (0.22-0.28) |
| Uzbekistan | 31291  (22778-42066) | 376.56  (273.59-501.81) | 73930  (53556-98726) | 410.03  (297.45-546.56) | 0.29  (0.26-0.31) |
| Vanuatu | 80  (59-109) | 122.57  (89.33-164.25) | 189  (140-254) | 133.58  (98.67-178.15) | 0.30  (0.28-0.31) |
| Venezuela (Bolivarian Republic of) | 37764  (26740-50847) | 419.23  (295.26-562.7) | 65970  (47379-89191) | 420.88  (303.13-567.37) | 0.01  (-0.02-0.05) |
| Viet Nam | 39947  (29166-54478) | 122.08  (90.53-163.9) | 79048  (58863-106024) | 140.72  (104.93-187.71) | 0.49  (0.48-0.5) |
| Yemen | 5574  (4103-7630) | 108.94  (80.37-146.52) | 16884  (12363-22995) | 112.77  (83.75-151.23) | 0.12  (0.11-0.13) |
| Zambia | 6060  (4498-8017) | 199.76  (148.42-265.42) | 16578  (12407-22036) | 202.35  (151.54-271.26) | 0.04  (0.04-0.05) |
| Zimbabwe | 18727  (13762-25648) | 447.21  (327.97-601.29) | 33325  (24620-44326) | 448.11  (330.96-594.61) | 0.01  (0-0.02) |

**Notes:**

ASR: age-standardized rate.

ASR for incidence is computed by direct standardization with global standard population in GBD 2019.

Net drifts are estimates derived from the age-period-cohort model and denotes overall annual percent change in incidence rate.

Parenthesis for all GBD health estimate indicates 95% uncertainty intervals; parenthesis for net drift indicates 95% confidence intervals.

| **Table S2.** The prevalence and age-standardized rate of uterine fibroids in 1990 and 2019, and the temporal trends from 1990 to 2019 at national level. | | | | | |
| --- | --- | --- | --- | --- | --- |
| Prevalence | | | | | |
| Nation | Cases in1990 | ASR in 1990  (per 100 000 persons) | Cases in 2019 | ASR in 2019  (per 100 000persons) | Net Drift  (%, per year) |
| Afghanistan | 90785  (68406-118610) | 2212.75  (1656.55-2918.35) | 299656 (228050-395079) | 2386.04  (1828.02-3138.24) | 0.26  (0.25-0.28) |
| Albania | 77083  (59529-97570) | 5770.83  (4441.78-7410.05) | 97218 (74151-124792) | 6102.93  (4688.63-7858.94) | 0.19  (0.19-0.2) |
| Algeria | 227575  (172832-300325) | 2613.39  (1984.72-3459.4) | 627970 (470422-831202) | 2772.8  (2091.44-3653.56) | 0.20  (0.19-0.22) |
| American Samoa | 552  (412-733) | 2980.51  (2223.84-3922.83) | 842 (638-1096) | 3193.42  (2414.65-4150.6) | 0.24  (0.22-0.26) |
| Andorra | 2242  (1692-2913) | 7578.57  (5723.03-9805.4) | 4586 (3411-6016) | 7869.56  (5947.36-10265.61) | 0.13  (0.11-0.15) |
| Angola | 180269  (137216-235988) | 5229.91  (3949.4-6855.16) | 635460 (484084-839005) | 5835.12  (4405.62-7654.26) | 0.38  (0.36-0.4) |
| Antigua and Barbuda | 2213  (1692-2908) | 7921.55  (6057.44-10247.31) | 4618 (3471-6040) | 8246.91  (6230.98-10798.8) | 0.13  (0.12-0.15) |
| Argentina | 936622  (706374-1216706) | 5759.62  (4320.68-7497.22) | 1546198 (1160061-2013254) | 6182.09  (4643.72-8050.07) | 0.24  (0.22-0.26) |
| Armenia | 148298  (113797-192890) | 9125.96  (6955.23-11843.79) | 180755 (136525-233819) | 9713.73  (7369.78-12548.06) | 0.22  (0.21-0.23) |
| Australia | 170720  (128319-223461) | 1852.65  (1394.94-2416.2) | 279694 (212861-364337) | 1915.86  (1449.97-2499.94) | 0.11  (0.1-0.12) |
| Austria | 387873  (303756-480265) | 8571.49  (6652.5-10563.75) | 439800 (341754-557355) | 7859.54  (6085.23-9888.39) | -0.31  (-0.37--0.25) |
| Azerbaijan | 279394  (216643-351747) | 8910.72  (6798.61-11398.25) | 593198 (447577-782201) | 9671.07  (7308.88-12783.73) | 0.28  (0.27-0.29) |
| Bahamas | 9603  (7250-12703) | 8228.77  (6214.74-10901.79) | 19433 (14688-25498) | 8402.94  (6357.71-11019.06) | 0.07  (0.07-0.08) |
| Bahrain | 4841  (3622-6447) | 2728.68  (2069.03-3609.43) | 19977 (14919-27006) | 2749.36  (2085.53-3665.75) | 0.03  (0.02-0.03) |
| Bangladesh | 1258786  (952693-1656721) | 3702.75  (2782.64-4841.87) | 3333502 (2512526-4398262) | 4120.99  (3127.06-5432.97) | 0.36  (0.33-0.39) |
| Barbados | 10721  (8088-13987) | 8243.15  (6212.8-10709.75) | 15757 (11992-20669) | 8371.55  (6361.79-10869.58) | 0.05  (0.04-0.07) |
| Belarus | 748320  (573289-955834) | 12401.81  (9485.3-15897.06) | 814044 (623938-1051579) | 12831.45  (9840.56-16553.98) | 0.12  (0.1-0.13) |
| Belgium | 440877  (335787-567325) | 7706.82  (5860.63-9870.01) | 535418 (403477-684182) | 7877  (5939.77-10077.02) | 0.06  (0.05-0.08) |
| Belize | 4671  (3527-6081) | 7994.81  (5992.1-10456.22) | 16568 (12444-21805) | 8447.39  (6325.67-11057.58) | 0.18  (0.17-0.2) |
| Benin | 85212  (64621-111705) | 5305.47  (3998.15-6867.55) | 257213 (195139-342094) | 5824.78  (4386.45-7668.32) | 0.32  (0.31-0.33) |
| Bermuda | 3135  (2374-4065) | 8656.84  (6594.19-11151.25) | 3686 (2776-4772) | 8764.47  (6579.17-11359.73) | 0.04  (0.04-0.05) |
| Bhutan | 7583  (5733-9897) | 3875.53  (2919.33-5046.85) | 15706 (11833-20722) | 4388.77  (3311.15-5788.82) | 0.43  (0.42-0.44) |
| Bolivia (Plurinational State of) | 277472  (208935-362757) | 11513.95  (8732.31-14983.01) | 658768 (497385-852502) | 11859.72  (8983.94-15274.86) | 0.1%  (0.09-0.11) |
| Bosnia and Herzegovina | 136485  (105036-176368) | 5610.4  (4303.49-7245.21) | 131123 (99526-168718) | 6190.2  (4750.48-7980.64) | 0.34%  (0.33-0.36) |
| Botswana | 47353  (36413-62192) | 10213.75  (7785.96-13259.44) | 132831 (100139-173398) | 11058.74  (8389.63-14327.46) | 0.33  (0.26-0.39) |
| Brazil | 2897273  (2266770-3693182) | 4501.37  (3514.69-5732.04) | 7886938 (6060574-10075679) | 6103.54  (4700.86-7799.73) | 1.03  (0.95-1.12) |
| Brunei Darussalam | 5362  (4028-7127) | 4714.61  (3563.22-6206.27) | 12917 (9750-17045) | 5074.94  (3846.42-6644.77) | 0.25  (0.25-0.26) |
| Bulgaria | 318547  (242690-421909) | 6140.03  (4684.31-8005.97) | 280688 (212837-366345) | 6404.47  (4919.1-8301.17) | 0.14  (0.13-0.15) |
| Burkina Faso | 169647  (130057-222959) | 5138.79  (3908.5-6749.62) | 464833 (353435-609570) | 5582.41  (4248.04-7232.81) | 0.28  (0.27-0.3) |
| Burundi | 75749  (57108-99272) | 3994.47  (3002.98-5196.26) | 159824 (121519-211159) | 3982.84  (3003.8-5216.4) | -0.01  (-0.02-0.01) |
| Cabo Verde | 6533  (5010-8582) | 5318.26  (4056.55-6947.05) | 15883 (11954-20904) | 5830.1  (4374.69-7583.54) | 0.31  (0.26-0.35) |
| Cambodia | 113582  (87054-148545) | 2826.18  (2151.79-3712.61) | 258758 (197049-344681) | 3087.05  (2347.09-4090.51) | 0.30  (0.29-0.31) |
| Cameroon | 204652  (156467-266223) | 5764.31  (4358.82-7579.66) | 647133 (486748-859960) | 5970.7  (4484.83-7807.94) | 0.11  (0.06-0.16) |
| Canada | 445114  (330847-590769) | 2832.31  (2109.11-3735.1) | 617593 (463770-807800) | 2841.51  (2124.95-3766.37) | 0  (-0.03-0.04) |
| Central African Republic | 53788  (40984-70732) | 5460.44  (4114.99-7116.81) | 110228 (83610-144266) | 5484.62  (4185.69-7142.33) | 0.02  (0.01-0.03) |
| Chad | 95916  (73321-125844) | 4718.73  (3581.06-6166.66) | 255036 (194982-338110) | 5166.46  (3915.45-6753.02) | 0.30  (0.25-0.35) |
| Chile | 360739  (271064-475457) | 5683.91  (4257.57-7449.03) | 676134 (510646-868212) | 6269.68  (4702.28-8113.48) | 0.33  (0.28-0.39) |
| China | 15191147  (11470081-20105563) | 2773.37  (2094.24-3656.62) | 26638185 (20623831-33706133) | 2934.66  (2261.33-3740.46) | 0.20  (0.17-0.23) |
| Colombia | 1167219  (871976-1528483) | 8938.73  (6679.71-11667.38) | 2415700 (1825631-3207505) | 8982.48  (6789.69-11909.73) | 0.02  (-0.01-0.04) |
| Comoros | 6800  (5150-8902) | 4347.96  (3282.2-5651.34) | 14304 (10888-18619) | 4391.52  (3342.68-5703.33) | 0.03  (0.02-0.04) |
| Congo | 46455  (35406-60645) | 5570.02  (4204.45-7296.37) | 135654 (102417-178488) | 5865.87  (4450.13-7596.36) | 0.18  (0.17-0.20) |
| Cook Islands | 230  (174-304) | 2989.54  (2259.53-3917.47) | 325 (247-423) | 3249.29  (2478.98-4277.22) | 0.29  (0.28-0.30) |
| Costa Rica | 108458  (80266-141975) | 9109.14  (6769.27-11996.12) | 255204 (188828-336790) | 9313.89  (6903.74-12295.05) | 0.07  (0.07-0.08) |
| Croatia | 183193  (138675-236384) | 6279.52  (4795.7-8022.45) | 170967 (132407-217211) | 6551.53  (5116.1-8286.57) | 0.15  (0.12-0.19) |
| Cuba | 461227  (349663-598442) | 8501.66  (6427.52-11045.23) | 625780 (475962-820009) | 8774.66  (6630.66-11397.24) | 0.11  (0.10-0.13) |
| Cyprus | 28286  (21427-36830) | 6929.44  (5246.35-9009.01) | 64938 (48453-82736) | 7483.16  (5609.67-9503.84) | 0.25  (0.19-0.32) |
| Czechia | 431161  (326056-565848) | 7099.17  (5367.74-9256.54) | 495253 (376223-656812) | 7382.99  (5649.62-9639.43) | 0.13  (0.06-0.20) |
| Côte d'Ivoire | 201978  (153297-264687) | 5358.13  (4015.72-6969.27) | 572355 (428620-759159) | 5887.28  (4418.42-7628.36) | 0.32  (0.28-0.35) |
| Democratic People's Republic of Korea | 227029  (170294-297940) | 2131.54  (1587.53-2803.39) | 342433 (256575-444356) | 2227.76  (1672.86-2904.66) | 0.15  (0.14-0.17) |
| Democratic Republic of the Congo | 686156  (521688-897496) | 5233.91  (3962.53-6812.04) | 1711312 (1277463-2242103) | 5498.88  (4131.26-7155.62) | 0.17  (0.12-0.21) |
| Denmark | 188768  (141324-247720) | 6250.69  (4685.95-8251.88) | 211544 (160001-275523) | 6290.56  (4710.51-8229.27) | 0.01  (-0.08-0.09) |
| Djibouti | 6091  (4671-8005) | 4176.41  (3191.94-5388.19) | 25128 (18804-34016) | 4580.77  (3488.47-6058.32) | 0.32  (0.31-0.34) |
| Dominica | 2358  (1795-3010) | 7899.85  (5933.77-10175.44) | 2997 (2301-3860) | 8208.97  (6266.24-10622.79) | 0.13  (0.11-0.15) |
| Dominican Republic | 216613  (163969-284250) | 7845.02  (5919.03-10319.42) | 456112 (346644-599568) | 8460.61  (6416.04-11104.07) | 0.26  (0.25-0.27) |
| Ecuador | 461788  (357664-591613) | 12040.32  (9365-15373.65) | 1041249 (816428-1310022) | 11931.59  (9355.79-15018.64) | -0.02  (-0.05-0) |
| Egypt | 588161  (443768-775919) | 2708.47  (2053.77-3527.08) | 1267121 (954972-1700959) | 2810.25  (2123.56-3736.21) | 0.13  (0.12-0.14) |
| El Salvador | 168192  (125208-221071) | 8657.28  (6434.78-11404.72) | 305098 (228017-400579) | 9012.75  (6715.59-11833.72) | 0.14  (0.12-0.16) |
| Equatorial Guinea | 8307  (6249-10866) | 5250.81  (3944.1-6824.94) | 30352 (23115-40076) | 6136.11  (4636.45-8060.93) | 0.54  (0.51-0.58) |
| Eritrea | 39268  (29547-51558) | 3853.93  (2891.12-5021.59) | 106225 (81269-139697) | 4105.24  (3131.01-5333.27) | 0.22  (0.2-0.23) |
| Estonia | 119208  (89436-154194) | 12598.93  (9492.99-16337.4) | 106728 (80777-140520) | 13127.11  (9989.26-17172.97) | 0.14  (0.10-0.18) |
| Eswatini | 28775  (21762-38094) | 10623.62  (8086.54-13829.56) | 53874 (40887-71324) | 10862.97  (8262.96-14241.33) | 0.08  (0.06-0.10) |
| Ethiopia | 685666  (521103-901574) | 4066.35  (3088.22-5305.76) | 1644242 (1248988-2157402) | 4362.62  (3310.93-5699.99) | 0.25  (0.24-0.26) |
| Fiji | 9302  (7003-12179) | 2890.38  (2170.86-3767.71) | 14507 (10935-19072) | 3138.14  (2371.26-4117.31) | 0.28  (0.26-0.30) |
| Finland | 215222  (160760-277230) | 7054.4  (5307.68-9076.6) | 226699 (173285-295685) | 7185.79  (5461.09-9363.25) | 0.04  (-0.01-0.08) |
| France | 2918698  (2215732-3758762) | 9036.34  (6866.84-11656.46) | 3661699 (2794979-4658741) | 9378.78  (7053.73-12055.7) | 0.13  (0.11-0.15) |
| Gabon | 19625  (14835-25571) | 5705.04  (4258.79-7453.67) | 50050 (38040-66143) | 6150.11  (4655.85-8060.24) | 0.26  (0.24-0.29) |
| Gambia | 16540  (12532-21700) | 5439  (4098.1-7068.15) | 47878 (36400-62995) | 5816.52  (4400.15-7585.15) | 0.23  (0.17-0.28) |
| Georgia | 283023  (214725-370747) | 9222.86  (6932.36-12130.68) | 248681 (194975-316297) | 10920.11  (8550.89-13693.02) | 0.58  (0.55-0.61) |
| Germany | 4602835  (3506754-5937397) | 9546.42  (7283.03-12352.95) | 5090972 (3882904-6530412) | 9867.65  (7463.96-12829.46) | 0.11  (0.01-0.20) |
| Ghana | 277796  (210749-366463) | 5247.04  (3970.85-6852.44) | 787175 (594658-1039021) | 5564.36  (4215.58-7267.14) | 0.20  (0.18-0.21) |
| Greece | 466826  (353567-608175) | 7804.39  (5888.61-10212.55) | 541884 (408525-696415) | 8107.1  (6177.58-10532.29) | 0.13  (0.12-0.14) |
| Greenland | 949  (718-1261) | 3788.67  (2860.71-5019.47) | 1188 (876-1537) | 3929.27  (2905.08-5102.66) | 0.13  (0.12-0.13) |
| Grenada | 2488  (1895-3212) | 7692.75  (5793.8-9949.13) | 4499 (3411-5829) | 8138.22  (6164.07-10574.88) | 0.20  (0.18-0.21) |
| Guam | 1800  (1369-2388) | 2990.69  (2262.27-3946.87) | 2721 (2040-3565) | 3244.71  (2428.4-4285.05) | 0.28  (0.27-0.29) |
| Guatemala | 224789  (165357-296454) | 8369.54  (6157.56-10935.54) | 697272 (517481-924250) | 8689.73  (6475.89-11561.94) | 0.12  (0.10-0.15) |
| Guinea | 116065  (87505-155653) | 5106.96  (3816.65-6784.63) | 248334 (188197-325860) | 5410.87  (4095.29-7071.62) | 0.20  (0.18-0.22) |
| Guinea-Bissau | 18242  (13931-24093) | 5211.05  (3970.34-6828.12) | 39748 (29983-52745) | 5429.05  (4108.15-7081.52) | 0.13%  (0.1-0.17) |
| Guyana | 23209  (17433-30564) | 7759.23  (5845.63-10144.14) | 32043 (24274-42253) | 8200.68  (6206.48-10876.23) | 0.19  (0.18-0.20) |
| Haiti | 173782  (132140-227852) | 7190.05  (5438.99-9312.78) | 432141 (326385-570689) | 7365.45  (5594.13-9608.19) | 0.08%  (0.06-0.09) |
| Honduras | 127477  (95545-168362) | 8501.81  (6303.05-11152.72) | 389696 (291496-515438) | 8981.73  (6684.45-11855.83) | 0.20  (0.17-0.22) |
| Hungary | 385699  (293064-496027) | 6135.25  (4679.52-7829.76) | 396231 (299663-514941) | 6290.35  (4811.77-8160.15) | 0.09  (0.08-0.09) |
| Iceland | 9056  (6888-11723) | 7184.31  (5442.21-9288.05) | 13779 (10438-17779) | 7119.21  (5354.24-9293.07) | -0.04  (-0.09-0.01) |
| India | 18417383  (13982575-24003201) | 5550.89  (4199.79-7172.27) | 49434384 (37473774-63869943) | 7200.81  (5446.3-9256.08) | 0.90  (0.87-0.94) |
| Indonesia | 2077889  (1582337-2737247) | 2616.78  (2001.77-3410.95) | 3699257 (2866465-4770779) | 2571.02  (1993.87-3315.1) | -0.05  (-0.08--0.02) |
| Iran (Islamic Republic of) | 600292  (454865-792220) | 3055.29  (2338.22-4014.13) | 1539304 (1170241-2040173) | 3100.41  (2367.85-4044.51) | 0.01  (-0.08-0.11) |
| Iraq | 156065  (118126-208248) | 2716.04  (2052.27-3603.32) | 499747 (378572-664044) | 2705.15  (2033.04-3563.84) | -0.02  (-0.05-0.02) |
| Ireland | 134175  (101064-175078) | 7648.34  (5728.23-9989.61) | 235814 (176826-305351) | 7815.58  (5900.89-10132.66) | 0.07  (0.06-0.08) |
| Israel | 174382  (130832-225630) | 7433.96  (5573.02-9646.17) | 363723 (269896-470146) | 7663.34  (5681.54-9927.54) | 0.1  (0.1-0.11) |
| Italy | 2235882  (1697057-2883683) | 6646.4  (5018.93-8678.05) | 2302834 (1797385-2890312) | 6405.97  (5021.72-8154.3) | -0.13  (-0.14--0.12) |
| Jamaica | 73799  (56737-96367) | 8001.33  (6079.58-10340.69) | 128410 (96369-167295) | 8430.3  (6341.38-10989.48) | 0.18  (0.17-0.19) |
| Japan | 5886244  (4490779-7654792) | 8080.59  (6204.49-10593.11) | 5552817 (4380019-7009039) | 8445.23  (6603.34-10737.5) | 0.15  (0.12-0.18) |
| Jordan | 30793  (23435-40574) | 2676.85  (2040.56-3534.34) | 138850 (104488-185079) | 2769.05  (2090.12-3690.41) | 0.12  (0.11-0.12) |
| Kazakhstan | 718095  (549185-929704) | 9199.69  (6996.44-11850.63) | 1021773 (772212-1355006) | 9792.83  (7451.52-13007.14) | 0.21  (0.19-0.24) |
| Kenya | 321726  (246350-419441) | 4586.21  (3489.95-5944.68) | 943791 (717779-1242697) | 4705.16  (3590.87-6098.2) | 0.09  (0.08-0.1) |
| Kiribati | 841  (642-1114) | 2790.31  (2119.45-3656.55) | 1732 (1296-2299) | 3094.51  (2315.01-4115.65) | 0.36  (0.33-0.38) |
| Kuwait | 17883  (13202-24034) | 2797.65  (2106.3-3639.62) | 83051 (60833-111487) | 2890.69  (2175.51-3802.9) | 0.12  (0.1-0.14) |
| Kyrgyzstan | 152724  (117421-197280) | 8719.64  (6662.07-11385.27) | 289291 (220844-371455) | 9111.08  (6918.77-11705.07) | 0.15  (0.08-0.21) |
| Lao People's Democratic Republic | 45267  (34431-59048) | 2895.54  (2219.33-3812.78) | 108887 (83236-141889) | 3199.72  (2446.34-4150.61) | 0.35  (0.34-0.35) |
| Latvia | 244273  (185809-318981) | 14983.98  (11383.49-19686.75) | 192921 (145441-251636) | 15612.81  (11803.14-20170.3) | 0.14  (0.09-0.20) |
| Lebanon | 38149  (29205-49798) | 2733.38  (2094.46-3560.22) | 84120 (63354-110535) | 2856.01  (2149.92-3744.76) | 0.15  (0.14-0.16) |
| Lesotho | 65189  (49655-85223) | 9947.74  (7531.39-12977.14) | 102030 (78133-134367) | 10762.47  (8190.56-14125.43) | 0.27  (0.27-0.28) |
| Liberia | 37578  (28791-49170) | 5392.74  (4089.82-6993.23) | 108503 (81883-143688) | 5697.88  (4312.22-7453.34) | 0.20  (0.18-0.20) |
| Libya | 33807  (25781-44426) | 2698.77  (2049.32-3561.7) | 106064 (79737-141057) | 2701.39  (2052.94-3562.92) | 0  (-0.01-0.01) |
| Lithuania | 214603  (163922-277490) | 10042.02  (7662.28-13017.73) | 187078 (143242-242569) | 10483.57  (7974.57-13609.59) | 0.13  (0.07-0.2) |
| Luxembourg | 22719  (17095-29218) | 9987.07  (7508.39-12841.6) | 40102 (30076-52435) | 10216.57  (7751.09-13461.92) | 0.06  (0.01-0.12) |
| Madagascar | 163071  (124677-216075) | 4134.72  (3136.95-5373.62) | 422878 (324112-554114) | 4126.15  (3180.49-5357.07) | 0  (-0.02-0.01) |
| Malawi | 139200  (105745-181417) | 4371.57  (3302.64-5667.06) | 285000 (214648-376661) | 4336.34  (3268.95-5727.72) | -0.03  (-0.04--0.02) |
| Malaysia | 243056  (184372-318895) | 3253.66  (2465.94-4232.13) | 570233 (433897-743674) | 3561.4  (2705.46-4639.48) | 0.32  (0.31-0.33) |
| Maldives | 2019  (1543-2654) | 2928.03  (2228.78-3867.81) | 7650 (5789-10266) | 3432.26  (2603.46-4533.12) | 0.54  (0.52-0.57) |
| Mali | 146515  (112538-192047) | 4837.73  (3703.83-6323.1) | 379411 (288324-498219) | 5369.08  (4027.17-7046.57) | 0.35  (0.33-0.38) |
| Malta | 14596  (10898-19001) | 6719.01  (5050.27-8731.41) | 18384 (14043-23756) | 7015.66  (5312.27-9097.01) | 0.13  (0.08-0.17) |
| Marshall Islands | 373  (280-493) | 2651.4  (1993.82-3459.94) | 798 (604-1052) | 2936.49  (2237.17-3833.24) | 0.35  (0.34-0.36) |
| Mauritania | 39247  (29874-51284) | 5484.34  (4155.18-7125.4) | 91916 (69689-120708) | 5893.4  (4456.74-7683.75) | 0.24  (0.19-0.28) |
| Mauritius | 17116  (13106-22496) | 3220.06  (2472.94-4220.18) | 26682 (20284-34638) | 3468.15  (2639.73-4523.47) | 0.25  (0.23-0.27) |
| Mexico | 3404800  (2574825-4480950) | 10720.57  (8123.28-14031.89) | 7121457 (5482672-9053667) | 10280.26  (7933.98-13052.4) | -0.15  (-0.16--0.13) |
| Micronesia (Federated States of) | 975  (743-1297) | 2812.09  (2139.23-3721.46) | 1439 (1092-1887) | 3026.62  (2284.85-3977.98) | 0.25  (0.25-0.26) |
| Monaco | 1553  (1191-2025) | 7820.73  (5931.7-10209.95) | 1885 (1434-2413) | 7949.26  (5975.61-10212.78) | 0.06  (0.04-0.07) |
| Mongolia | 63302  (48860-80557) | 8850.03  (6734.58-11315.44) | 177250 (134309-233699) | 9399.85  (7145.77-12343.64) | 0.21  (0.19-0.22) |
| Montenegro | 20087  (15318-25875) | 6095.81  (4651.36-7845.34) | 24419 (18414-32003) | 6444.14  (4920.26-8400.85) | 0.19  (0.14-0.23) |
| Morocco | 259106  (196553-341875) | 2587.33  (1961.38-3416.63) | 517710 (389522-683149) | 2669.34  (2016.1-3514.49) | 0.11  (0.1-0.12) |
| Mozambique | 198824  (150695-261719) | 4062.67  (3066.09-5285.07) | 445977 (339039-585459) | 4383.17  (3305.01-5701.9) | 0.27  (0.25-0.28) |
| Myanmar | 485930  (368741-639918) | 2883.15  (2197.87-3779.09) | 977991 (746714-1275701) | 3229.16  (2467.61-4210.32) | 0.39  (0.37-0.41) |
| Namibia | 50179  (38189-64626) | 10136.92  (7734.69-13003.64) | 113761 (86910-147679) | 10499.85  (8054.39-13557.99) | 0.12  (0.11-0.14) |
| Nauru | 111  (84-145) | 2901.95  (2208.45-3760.3) | 146 (110-193) | 3134.82  (2373.57-4115.61) | 0.27  (0.26-0.28) |
| Nepal | 197831  (150091-257007) | 2784.91  (2097.56-3608.13) | 426054 (327917-540143) | 2846.44  (2184.52-3625.02) | 0.06  (0-0.12) |
| Netherlands | 651474  (493571-846232) | 7616.29  (5769.92-9890.79) | 789684 (599180-1019600) | 7706.94  (5841.6-9987.19) | 0.04  (0.03-0.04) |
| New Zealand | 37323  (28248-48728) | 2058.32  (1556.21-2691.78) | 49380 (39027-61147) | 1830.67  (1451.65-2274.51) | -0.41  (-0.47--0.36) |
| Nicaragua | 103821  (77678-136480) | 8606.47  (6441.4-11425.5) | 283975 (212261-373630) | 9012.75  (6715.59-11833.72) | 0.16  (0.15-0.17) |
| Niger | 119855  (90627-157810) | 4838.96  (3631.82-6305.65) | 349351 (263413-464668) | 5158.18  (3901.58-6768.94) | 0.22  (0.21-0.23) |
| Nigeria | 634968  (1249762-2145743) | 5703.92  (4332.55-7376.87) | 4783124 (3664753-6241388) | 5935.27  (4517.74-7655.73) | 0.13  (0.1-0.16) |
| Niue | 29  (22-38) | 2935.12  (2225.66-3882.92) | 28 (22-37) | 3213.13  (2426.36-4225.2) | 0.31  (0.29-0.33) |
| North Macedonia | 61304  (46855-78691) | 5835.58  (4459.57-7496.36) | 81946 (62458-106011) | 6207.77  (4759.21-8046.93) | 0.21  (0.21-0.22) |
| Northern Mariana Islands | 619  (457-827) | 2963.85  (2229.97-3842.85) | 756 (570-972) | 3125.63  (2358.42-4113.17) | 0.19  (0.18-0.19) |
| Norway | 118578  (88522-156188) | 5104.77  (3795.01-6733.41) | 158763 (118171-210744) | 5148.14  (3818.58-6809.43) | -0.02  (-0.07-0.04) |
| Oman | 13251  (9940-17609) | 2538.94  (1926.27-3356.48) | 46682 (34607-62942) | 2756.83  (2079.78-3636.59) | 0.29  (0.27-0.31) |
| Pakistan | 1629398  (1227830-2139474) | 4469.16  (3366.06-5841.8) | 3990945 (3050636-5264799) | 4611.73  (3503.77-5991.79) | 0.11  (0.07-0.15) |
| Palau | 208  (156-274) | 2973.64  (2239.89-3891.24) | 330 (247-439) | 3210.31  (2394.72-4235.58) | 0.27  (0.26-0.28) |
| Palestine | 16517  (12547-21898) | 2568.44  (1944.48-3402.78) | 52743 (39691-69987) | 2647.93  (1992.08-3502.99) | 0.10  (0.08-0.12) |
| Panama | 80574  (59551-105656) | 8483.67  (6228.89-11209.83) | 190751 (140382-245087) | 9003.86  (6637.01-11575.48) | 0.20  (0.2-0.21) |
| Papua New Guinea | 39523  (29663-53037) | 2649.93  (1977.23-3555.21) | 121505 (91533-161469) | 2883.58  (2186.16-3830.36) | 0.29  (0.27-0.31) |
| Paraguay | 71114  (54083-93196) | 4760.94  (3581.62-6186.85) | 170491 (127535-226091) | 5095.86  (3806.76-6776.48) | 0.24  (0.23-0.24) |
| Peru | 953119  (717387-1249349) | 11352.26  (8509.73-14950.4) | 2150494 (1644439-2845632) | 11923.45  (9116.7-15758.25) | 0.17  (0.15-0.2) |
| Philippines | 1020448  (782946-1338423) | 4129.1  (3152.11-5400.54) | 2429523 (1863636-3178404) | 4626.53  (3541.57-6029.41) | 0.39  (0.38-0.4) |
| Poland | 1038185  (785278-1333700) | 4928.56  (3777.17-6327.14) | 974112 (798881-1190636) | 4164.04  (3430.22-5067.24) | -0.58  (-0.65--0.52) |
| Portugal | 339196  (254866-439692) | 5905.31  (4428.37-7702.59) | 388825 (299154-493885) | 5481.91  (4208.66-6901.97) | -0.27  (-0.35--0.20) |
| Puerto Rico | 161313  (121329-209717) | 8472.86  (6363.81-11004.01) | 190156 (145020-243958) | 8769.75  (6659.13-11377.53) | 0.12  (0.11-0.13) |
| Qatar | 3596  (2657-4908) | 2735.62  (2077.19-3588.27) | 25849 (18973-34678) | 2812.82  (2109.69-3679.71) | 0.10  (0.08-0.12) |
| Republic of Korea | 813620  (614697-1077421) | 3472.86  (2628.2-4590.86) | 1189125 (901217-1560668) | 3757.38  (2828.17-4963.45) | 0.27  (0.22-0.32) |
| Republic of Moldova | 286527  (219456-369542) | 11784.04  (8927.5-15201.66) | 306031 (232009-394178) | 12504.79  (9431.5-16014.81) | 0.21  (0.19-0.22) |
| Romania | 703415  (542764-894839) | 5448.22  (4166.33-6902.43) | 695850 (527616-886887) | 5741.96  (4360.13-7292.99) | 0.19  (0.17-0.21) |
| Russian Federation | 12008764  (9235487-15361683) | 13457.01  (10382.59-17290.57) | 12865156 (9891345-16488306) | 13336.71  (10236.11-16959.87) | -0.03  (-0.04--0.01) |
| Rwanda | 102985  (78073-137100) | 4290.83  (3211.52-5661.45) | 235786 (179173-310765) | 4438.08  (3362.99-5820.31) | 0.12  (0.11-0.12) |
| Saint Kitts and Nevis | 1326  (1009-1728) | 8201.79  (6170.43-10677.59) | 3052 (2304-3999) | 8520.67  (6405.77-11181.67) | 0.10 3(0.13-0.14) |
| Saint Lucia | 4107  (3099-5416) | 7918.04  (5900.7-10441.88) | 8804 (6661-11447) | 8240  (6224.95-10753.46) | 0.13  (0.12-0.14) |
| Saint Vincent and the Grenadines | 3038  (2306-4003) | 7711.14  (5763.93-10192.12) | 5107 (3823-6576) | 8208.09  (6123.96-10637.8) | 0.21  (0.20-0.23) |
| Samoa | 1687  (1270-2220) | 3006.71  (2262.49-3957.04) | 2834 (2170-3715) | 3238.18  (2468.03-4252.98) | 0.26  (0.25-0.26) |
| San Marino | 980  (747-1258) | 7746.62  (5842.93-10063.22) | 1687 (1263-2180) | 7870.23  (5963.42-10179.1) | 0.06  (0.04-0.08) |
| Sao Tome and Principe | 2043  (1569-2657) | 5265.51  (4001.53-6839.33) | 4811 (3644-6379) | 5591.39  (4252.41-7382.86) | 0.20  (0.15-0.24) |
| Saudi Arabia | 120244  (90794-158692) | 2562.77  (1937.13-3378.11) | 509266 (376683-683929) | 2756.63  (2087.37-3649.17) | 0.25  (0.24-0.26) |
| Senegal | 137474  (105284-180599) | 5511.36  (4185.13-7211.22) | 325723 (247158-425439) | 5593.84  (4243.68-7246.99) | 0.05  (0.01-0.08) |
| Serbia | 313194  (238729-400835) | 5789.57  (4431.11-7456.73) | 322039 (244797-418821) | 6202.21  (4775.01-8044.65) | 0.24  (0.23-0.26) |
| Seychelles | 969  (740-1269) | 3220.97  (2453.84-4238.93) | 1934 (1468-2551) | 3440.76  (2634.88-4569.7) | 0.23  (0.21-0.24) |
| Sierra Leone | 71811  (54593-93895) | 5352.12  (4079.77-6957.11) | 177983 (134872-236118) | 5814.49  (4372.2-7645.06) | 0.28  (0.26-0.3) |
| Singapore | 81446  (61451-107798) | 4517.85  (3441.4-5971.69) | 181600 (138006-239774) | 4903.7  (3754.54-6474.9) | 0.28  (0.27-0.29) |
| Slovakia | 208916  (157734-269317) | 7286.13  (5508.03-9399.21) | 265015 (199153-350116) | 7613.03  (5807.98-9993.28) | 0.15  (0.09-0.21) |
| Slovenia | 66017  (50351-85537) | 5834.5  (4449.53-7515.9) | 79102 (59583-101911) | 6197.78  (4741.96-7965.42) | 0.22  (0.19-0.24) |
| Solomon Islands | 2874  (2187-3764) | 2683.4  (2032.92-3480.98) | 8094 (6098-10660) | 2949.08  (2232.73-3864.72) | 0.33  (0.31-0.34) |
| Somalia | 102125  (76770-135074) | 4073.66  (3073.11-5345.35) | 274704 (209067-358951) | 4140.67  (3149.03-5389.64) | 0.06  (0.05-0.07) |
| South Africa | 1845825  (1416976-2406792) | 11894.41  (9061.02-15295.2) | 3580611 (2741946-4645069) | 11956.13  (9148.84-15407.72) | 0.02  (0.01-0.03) |
| South Sudan | 71676  (54831-94018) | 4103.42  (3105.16-5372.14) | 142804 (108143-187633) | 4160.99  (3126.38-5437.21) | 0.05  (0.04-0.06) |
| Spain | 2071861  (1564586-2683135) | 9801.54  (7363.11-12818.4) | 3104157 (2333645-4018108) | 10103.9  (7615.46-13055.23) | 0.11  (0.07-0.14) |
| Sri Lanka | 244404  (186613-322463) | 3043.04  (2323.16-3993.22) | 410638 (314711-539256) | 3270.34  (2503.15-4315.36) | 0.25  (0.24-0.25) |
| Sudan | 154680  (117372-204755) | 2198.14  (1671.42-2899.78) | 405063 (301750-544000) | 2434.6  (1836.09-3228.32) | 0.36  (0.34-0.37) |
| Suriname | 13073  (9890-16957) | 8076.15  (6068.28-10494.73) | 27205 (20614-35245) | 8547.41  (6458.49-11105.61) | 0.19  (0.19-0.2) |
| Sweden | 277908  (209020-356408) | 5642.34  (4241.75-7295.06) | 311486 (238949-403764) | 5386.52  (4111.02-7027.67) | -0.16  (-0.25--0.08) |
| Switzerland | 311912  (234575-398789) | 7617.78  (5722.79-9722.47) | 418039 (313343-530678) | 7663.3  (5770.7-9753.83) | 0.02  (0.01-0.03) |
| Syrian Arab Republic | 104012  (78435-137445) | 2623.59  (1974.89-3454.33) | 206325 (155421-271863) | 2695.86  (2044.2-3558.57) | 0.09  (0.08-0.1) |
| Taiwan (Province of China) | 241117  (178969-316918) | 2395.56  (1779.98-3138.25) | 415397 (308706-546858) | 2657.13  (1979.82-3511.92) | 0.36  (0.35-0.36) |
| Tajikistan | 150974  (115193-193014) | 8680.45  (6564-11263.73) | 370874 (282630-480250) | 8822.38  (6694.09-11418.29) | 0.05  (0.04-0.07) |
| Thailand | 862167  (659871-1146478) | 3110.65  (2388.61-4101.22) | 1564938 (1190846-2050530) | 3418.21  (2599.93-4501.19) | 0.32  (0.31-0.34) |
| Timor-Leste | 8357  (6390-10995) | 2829.21  (2158.25-3712.62) | 15615 (11998-20456) | 3083.63  (2364.85-4060.25) | 0.30  (0.29-0.31) |
| Togo | 63432  (48192-83840) | 5195.23  (3909.66-6829.39) | 188426 (141631-247190) | 5535.42  (4180.88-7198.44) | 0.22  (0.21-0.23) |
| Tokelau | 19  (14-25) | 2832.83  (2135.66-3725.29) | 20 (16-27) | 3151.51  (2391.54-4151.28) | 0.37  (0.36-0.38) |
| Tonga | 1077  (813-1416) | 2989.54  (2235.31-3917.17) | 1465 (1125-1926) | 3196.9  (2450.5-4196.66) | 0.24  (0.22-0.26) |
| Trinidad and Tobago | 42848  (32282-56181) | 8109.02  (6089.37-10558.63) | 70280 (53105-91246) | 8494.01  (6431.21-11088.28) | 0.16  (0.14-0.18) |
| Tunisia | 87015  (66906-115749) | 2632.43  (2010.96-3474.92) | 192261 (144607-253992) | 2789.69  (2109.02-3679.35) | 0.19  (0.14-0.24) |
| Turkey | 596316  (453797-781720) | 2427.69  (1853.18-3157.87) | 1242133 (931429-1656684) | 2606.02  (1958.03-3459.4) | 0.25  (0.23-0.26) |
| Turkmenistan | 117552  (90362-151177) | 8966.35  (6774.07-11599.43) | 241860 (182848-315590) | 9492.67  (7184.72-12370.09) | 0.19  (0.14-0.23) |
| Tuvalu | 130  (99-170) | 2796.71  (2122.33-3629.79) | 166 (126-218) | 3085.81  (2342.62-4061.92) | 0.34  (0.33-0.35) |
| Uganda | 215844  (163710-283833) | 4131.68  (3115.28-5385.36) | 578799 (443128-760703) | 4244.39  (3239.84-5514.53) | 0.09  (0.08-0.1) |
| Ukraine | 4197558  (3228690-5346590) | 13114.41  (10041.81-16671.51) | 3946347 (3006927-5054668) | 13122.52  (10025.23-16783.37) | 0  (-0.01-0.01) |
| United Arab Emirates | 13446  (9817-18175) | 2604.39  (1962.68-3404.28) | 105698 (76265-142913) | 2764.51  (2108.95-3663.37) | 0.21  (0.2-0.21) |
| United Kingdom | 2107557  (1596908-2731369) | 6456.88  (4873.75-8374.15) | 2333441 (1805643-2950859) | 5745.72  (4436.32-7329.44) | -0.40  (-0.43--0.36) |
| United Republic of Tanzania | 371132  (277717-490458) | 4346.91  (3268.51-5712.78) | 954560 (722191-1248771) | 4486.13  (3391.02-5870.97) | 0.11  (0.1-0.12) |
| United States of America | 7754354  (6064417-9778755) | 5447.55  (4269.79-6865.57) | 11672754 (9185031-14636493) | 6169.48  (4828.84-7767.28) | 0.42  (0.36-0.48) |
| United States Virgin Islands | 4842  (3624-6363) | 8218.64  (6197.21-10771.86) | 5436 (4118-7066) | 8549.6  (6482.81-11139.51) | 0.13  (0.12-0.15) |
| Uruguay | 93398  (70931-121479) | 5727.03  (4340.26-7498.27) | 123034 (93245-159064) | 6236.03  (4699.57-8114.75) | 0.29  (0.28-0.31) |
| Uzbekistan | 621152  (472235-791039) | 8525.81  (6435.67-10926.29) | 1583997 (1208600-2038099) | 9303.19  (7078.24-12025.63) | 0.30  (0.29-0.31) |
| Vanuatu | 1445  (1072-1940) | 2732.91  (2021.79-3624.88) | 3627 (2772-4764) | 2969.37  (2253.8-3892.28) | 0.29  (0.27-0.31) |
| Venezuela (Bolivarian Republic of) | 677005  (502234-889798) | 9254.56  (6884.68-12220.77) | 1491971 (1114452-1963375) | 9302.28  (6961.58-12197.21) | 0.02  (0.01-0.02) |
| Viet Nam | 749399  (568737-990608) | 2719.83  (2069.96-3576.83) | 1818147 (1391242-2399946) | 3153.85  (2410.18-4147.47) | 0.51  (0.5-0.53) |
| Yemen | 98843  (74021-132634) | 2379.42  (1789.6-3185.58) | 304130 (228668-404381) | 2459.35  (1860.42-3233.88) | 0.12  (0.11-0.13) |
| Zambia | 110250  (84056-145741) | 4495.47  (3392.27-5921.43) | 296982 (225907-394506) | 4569.58  (3449.16-5969.55) | 0.05  (0.05-0.06) |
| Zimbabwe | 346795  (263788-455768) | 10473.5  (7912.95-13703.32) | 649996 (492311-850638) | 10478.5  (7979.09-13577.94) | 0  (-0.01-0.02) |

**Notes:**

ASR: age-standardized rate.

ASR for prevalence is computed by direct standardization with global standard population in GBD 2019.

Net drifts are estimates derived from the age-period-cohort model and denotes overall annual percent change in prevalence rate.

Parenthesis for all GBD health estimate indicates 95% uncertainty intervals; parenthesis for net drift indicates 95% confidence intervals.

| **Table S3.** The years lived with disability and age-standardized rate of uterine fibroids in 1990 and 2019, and the temporal trends from 1990 to 2019 at national level. | | | | | |  |
| --- | --- | --- | --- | --- | --- | --- |
| YLDs | | | | | |  |
| Nation | Cases in1990 | ASR in 1990  (per 100 000 persons) | Cases in 2019 | ASR in 2019  (per 100 000persons) | Net Drift  (%, per year) | |
| Afghanistan | 515  (246-961) | 12.55  (6.02-23.32) | 1688  (813-3111) | 13.33  (6.41-24.47) | 0.21  (0.18-0.25) | |
| Albania | 434  (201-841) | 32.32  (14.8-61.59) | 535  (243-1021) | 33.77  (15.48-65.02) | 0.15  (0.14-0.17) | |
| Algeria | 1298  (598-2437) | 14.85  (6.88-28.1) | 3521  (1643-6511) | 15.51  (7.23-28.53) | 0.15  (0.13-0.17) | |
| American Samoa | 3  (1-6) | 16.9  (7.78-31.67) | 5  (2-9) | 17.87  (8.22-33.88) | 0.19  (0.17-0.21) | |
| Andorra | 12  (6-23) | 41.21  (18.9-77.57) | 25  (11-48) | 42.7  (19.66-82.99) | 0.12  (0.1-0.14) | |
| Angola | 1031  (492-1939) | 29.71  (14.05-56.33) | 3591  (1671-6692) | 32.75  (15.12-61.67) | 0.34  (0.32-0.36) | |
| Antigua and Barbuda | 12  (6-24) | 44.12  (21.02-84.7) | 26  (12-49) | 45.65  (21.42-87.39) | 0.11  (0.1-0.13) | |
| Argentina | 5185  (2375-10008) | 31.91  (14.65-61.58) | 8487  (3900-16310) | 33.97  (15.65-65.25) | 0.21  (0.19-0.23) | |
| Armenia | 821  (378-1578) | 50.38  (23.33-96.86) | 992  (456-1887) | 53.48  (24.67-102.02) | 0.21  (0.2-0.22) | |
| Australia | 934  (423-1786) | 10.13  (4.59-19.37) | 1524  (687-2980) | 10.49  (4.72-20.48) | 0.11  (0.07-0.15) | |
| Austria | 2094  (955-3900) | 46.38  (21.21-85.9) | 2374  (1093-4503) | 42.62  (19.59-79.89) | -0.3  (-0.36--0.24) | |
| Azerbaijan | 1577  (733-3063) | 50.01  (23.43-96.77) | 3297  (1521-6343) | 53.75  (24.84-103.78) | 0.25  (0.23-0.26) | |
| Bahamas | 54  (25-102) | 45.9  (21.47-87.58) | 108  (51-202) | 46.71  (22.23-88.3) | 0.06  (0.05-0.07) | |
| Bahrain | 28  (13-52) | 15.43  (7.32-28.49) | 110  (51-209) | 15.1  (7.03-28.32) | -0.07  (-0.09--0.05) | |
| Bangladesh | 7275  (3487-13627) | 21.24  (10.12-39.35) | 18610  (8636-35349) | 22.95  (10.75-43.26) | 0.26  (0.22-0.3) | |
| Barbados | 59  (28-116) | 45.54  (21.49-89.29) | 86  (40-161) | 45.97  (21.32-85.83) | 0.03  (0.01-0.05) | |
| Belarus | 4093  (1890-7805) | 68.01  (31.49-131) | 4442  (2077-8447) | 70.36  (32.6-135.48) | 0.12  (0.1-0.14) | |
| Belgium | 2383  (1091-4528) | 41.77  (18.95-79.12) | 2883  (1331-5565) | 42.61  (19.53-81.64) | 0.06  (0.04-0.07) | |
| Belize | 27  (13-50) | 45.08  (21.56-84.32) | 93  (44-175) | 47.16  (22.41-88.48) | 0.15  (0.13-0.17) | |
| Benin | 489  (233-911) | 30.17  (14.2-56.4) | 1475  (702-2807) | 33.12  (15.77-63.18) | 0.32  (0.29-0.34) | |
| Bermuda | 17  (8-33) | 47.95  (22.85-89.61) | 20  (10-38) | 48.4  (22.74-90.51) | 0.03  (0.02-0.04) | |
| Bhutan | 44  (21-81) | 22.32  (10.41-41.44) | 89  (42-167) | 24.87  (11.57-46.18) | 0.37  (0.36-0.38) | |
| Bolivia (Plurinational State of) | 1586  (760-3008) | 65.37  (31.33-124.27) | 3683  (1765-7028) | 66.09  (31.37-125.81) | 0.04  (0.03-0.05) | |
| Bosnia and Herzegovina | 755  (349-1435) | 31.03  (14.26-58.96) | 716  (324-1364) | 34.08  (15.56-66.07) | 0.32  (0.3-0.34) | |
| Botswana | 272  (131-510) | 58.29  (28.05-109.58) | 730  (341-1396) | 60.51  (28.57-115.1) | 0.18  (0.1-0.26) | |
| Brazil | 16417  (7814-30628) | 25.3  (12.05-46.72) | 43723  (20453-81167) | 33.86  (15.81-62.66) | 0.99  (0.9-1.08) | |
| Brunei Darussalam | 31  (14-58) | 26.77  (12.43-50.78) | 73  (34-139) | 28.58  (13.2-53.96) | 0.23  (0.21-0.25) | |
| Bulgaria | 1747  (798-3326) | 33.84  (15.58-64.39) | 1534  (710-2908) | 35.31  (16.07-67.75) | 0.15  (0.13-0.16) | |
| Burkina Faso | 959  (457-1815) | 28.89  (13.64-54.37) | 2675  (1284-5020) | 31.91  (15.19-59.73) | 0.35  (0.32-0.37) | |
| Burundi | 432  (203-803) | 22.61  (10.6-41.94) | 908  (418-1680) | 22.48  (10.46-41.96) | -0.02  (-0.05-0.01) | |
| Cabo Verde | 37  (17-69) | 30.19  (14.21-56.43) | 89  (41-168) | 32.58  (15.03-61.14) | 0.25  (0.19-0.31) | |
| Cambodia | 660  (316-1229) | 16.34  (7.9-29.81) | 1470  (683-2755) | 17.5  (8.12-32.85) | 0.23  (0.2-0.27) | |
| Cameroon | 1161  (548-2184) | 32.52  (15.28-61.25) | 3688  (1776-7003) | 33.75  (16.17-63.85) | 0.13  (0.11-0.15) | |
| Canada | 2443  (1108-4723) | 15.54  (7.02-29.99) | 3369  (1533-6549) | 15.59  (7.01-30.44) | 0  (-0.03-0.04) | |
| Central African Republic | 305  (146-568) | 30.81  (14.65-58.07) | 627  (294-1172) | 30.98  (14.56-58.17) | 0.02  (0-0.04) | |
| Chad | 552  (266-1046) | 27.01  (12.94-51.2) | 1470  (699-2740) | 29.49  (13.99-55.36) | 0.29  (0.24-0.34) | |
| Chile | 1987  (917-3827) | 31.21  (14.39-60.06) | 3677  (1655-6959) | 34.19  (15.41-65.28) | 0.31  (0.27-0.35) | |
| China | 85926  (40081-160609) | 15.61  (7.3-29.31) | 146550  (68793-274820) | 16.21  (7.58-30.38) | 0.13  (0.09-0.18) | |
| Colombia | 6515  (3067-12297) | 49.58  (23.33-93.28) | 13194  (6129-24714) | 49.11  (22.86-92.53) | -0.03  (-0.06-0.01) | |
| Comoros | 39  (19-73) | 24.93  (11.8-46.56) | 82  (39-152) | 24.95  (11.83-46.6) | 0  (-0.02-0.02) | |
| Congo | 267  (126-504) | 31.84  (15.2-60.1) | 766  (364-1442) | 32.95  (15.71-62.25) | 0.12  (0.1-0.13) | |
| Cook Islands | 1  (1-2) | 16.89  (7.96-31.19) | 2  (1-3) | 18.08  (8.5-34.06) | 0.24  (0.23-0.25) | |
| Costa Rica | 602  (280-1139) | 50.24  (23.48-95.38) | 1393  (647-2726) | 50.85  (23.63-99.7) | 0.04  (0.03-0.05) | |
| Croatia | 1000  (451-1930) | 34.41  (15.61-67.14) | 929  (434-1745) | 35.9  (16.96-68.85) | 0.15  (0.12-0.19) | |
| Cuba | 2551  (1212-4865) | 46.95  (22.26-89.34) | 3427  (1596-6546) | 48.33  (22.83-93.27) | 0.1  (0.09-0.12) | |
| Cyprus | 154  (70-291) | 37.75  (17.22-71.44) | 351  (160-662) | 40.46  (18.46-76.66) | 0.23  (0.17-0.29) | |
| Czechia | 2360  (1078-4575) | 39.02  (17.77-75.87) | 2689  (1229-5139) | 40.43  (18.57-77.7) | 0.12  (0.05-0.2) | |
| Côte d'Ivoire | 1161  (557-2147) | 30.46  (14.38-56.56) | 3279  (1551-6267) | 33.44  (15.97-63.72) | 0.31  (0.26-0.37) | |
| Democratic People's Republic of Korea | 1294  (600-2406) | 12.17  (5.66-22.64) | 1918  (889-3556) | 12.54  (5.86-23.39) | 0.11  (0.08-0.14) | |
| Democratic Republic of the Congo | 3968  (1890-7311) | 30.07  (14.47-55.35) | 9753  (4582-18188) | 31.14  (14.68-58.05) | 0.12  (0.1-0.14) | |
| Denmark | 1018  (461-1911) | 33.81  (15.36-63.83) | 1137  (517-2202) | 33.98  (15.39-65.63) | 0  (-0.09-0.09) | |
| Djibouti | 35  (17-66) | 23.84  (11.18-45.23) | 143  (67-272) | 25.98  (12.16-48.78) | 0.3  (0.29-0.31) | |
| Dominica | 13  (6-25) | 44.3  (20.93-83.89) | 17  (8-31) | 45.75  (21.36-86.69) | 0.11  (0.09-0.12) | |
| Dominican Republic | 1234  (590-2335) | 44.37  (21.07-84.79) | 2535  (1190-4726) | 46.96  (22.06-87.4) | 0.2  (0.18-0.21) | |
| Ecuador | 2615  (1258-4970) | 67.69  (32.44-127.24) | 5746  (2712-10799) | 65.75  (31.06-123.46) | -0.09  (-0.13--0.05) | |
| Egypt | 3354  (1575-6370) | 15.38  (7.25-29.38) | 7044  (3218-13269) | 15.57  (7.13-29.12) | 0.04  (0.02-0.06) | |
| El Salvador | 934  (435-1790) | 47.89  (22.24-91.64) | 1661  (776-3087) | 49.07  (23.02-91.5) | 0.09  (0.08-0.1) | |
| Equatorial Guinea | 48  (23-90) | 30.08  (14.24-56.26) | 169  (80-321) | 33.82  (15.76-65.15) | 0.41  (0.37-0.45) | |
| Eritrea | 227  (106-420) | 22.1  (10.34-41.05) | 607  (288-1142) | 23.33  (10.99-43.76) | 0.19  (0.16-0.22) | |
| Estonia | 652  (302-1242) | 69.11  (31.95-131.9) | 582  (269-1100) | 72.03  (33.29-138.61) | 0.14  (0.09-0.19) | |
| Eswatini | 164  (78-312) | 60.11  (28.43-113.02) | 295  (140-575) | 59.14  (27.94-115.18) | -0.06  (-0.07--0.05) | |
| Ethiopia | 3934  (1863-7204) | 23.13  (11.04-42.67) | 9359  (4451-17329) | 24.59  (11.55-46.07) | 0.21  (0.19-0.23) | |
| Fiji | 53  (25-100) | 16.26  (7.65-30.64) | 81  (38-154) | 17.58  (8.27-33.19) | 0.27  (0.25-0.29) | |
| Finland | 1169  (539-2195) | 38.45  (17.78-72.09) | 1225  (557-2325) | 39.1  (17.56-74.41) | 0.03  (-0.01-0.08) | |
| France | 15746  (7177-29854) | 48.84  (22.45-92.76) | 19704  (8983-37340) | 50.66  (23.07-96.25) | 0.13  (0.11-0.15) | |
| Gabon | 114  (54-212) | 32.92  (15.66-61.42) | 285  (135-534) | 34.9  (16.57-65.82) | 0.21  (0.18-0.24) | |
| Gambia | 96  (45-181) | 31.29  (14.71-58.03) | 277  (132-512) | 33.36  (15.95-61.58) | 0.22  (0.17-0.26) | |
| Georgia | 1574  (734-2997) | 51.37  (24.08-98.14) | 1370  (642-2528) | 60.51  (28.43-111.71) | 0.56  (0.53-0.59) | |
| Germany | 24892  (11428-47559) | 51.79  (23.57-98.71) | 27384  (12526-51944) | 53.38  (24.25-102.58) | 0.1  (0-0.2) | |
| Ghana | 1610  (756-3007) | 30.16  (14.18-56.42) | 4505  (2119-8532) | 31.65  (14.88-59.68) | 0.16  (0.14-0.18) | |
| Greece | 2525  (1146-4858) | 42.3  (19.12-82.01) | 2920  (1326-5577) | 43.86  (19.99-83.95) | 0.12  (0.11-0.14) | |
| Greenland | 5  (2-10) | 20.87  (9.52-40.78) | 7  (3-13) | 21.63  (9.9-41.32) | 0.12  (0.11-0.14) | |
| Grenada | 14  (7-27) | 43.24  (20.39-81.86) | 25  (12-47) | 45.45  (21.41-86.98) | 0.18  (0.14-0.21) | |
| Guam | 10  (5-19) | 16.8  (7.8-31.87) | 15  (7-28) | 18.15  (8.44-34.17) | 0.27  (0.25-0.28) | |
| Guatemala | 1267  (603-2434) | 46.91  (22.35-89.19) | 3853  (1781-7189) | 47.76  (22.24-90.38) | 0.05  (0.04-0.07) | |
| Guinea | 670  (325-1271) | 29.31  (13.96-55.98) | 1422  (685-2696) | 30.73  (14.52-57.51) | 0.16  (0.15-0.18) | |
| Guinea-Bissau | 105  (49-195) | 29.73  (13.98-55.54) | 229  (108-426) | 30.98  (14.62-57.43) | 0.13  (0.08-0.19) | |
| Guyana | 133  (65-251) | 44  (21.42-83.13) | 179  (86-341) | 45.75  (22.16-87.67) | 0.13  (0.12-0.15) | |
| Haiti | 1008  (490-1875) | 41.49  (20.07-77.08) | 2466  (1192-4722) | 41.73  (20.05-78.98) | 0.01  (0-0.03) | |
| Honduras | 715  (333-1368) | 47.37  (22.11-91.35) | 2151  (1005-4102) | 49.37  (23.01-94.21) | 0.15  (0.13-0.17) | |
| Hungary | 2110  (945-4000) | 33.73  (15.27-64.23) | 2160  (973-4109) | 34.6  (15.86-66.48) | 0.09  (0.07-0.1) | |
| Iceland | 49  (23-94) | 38.98  (17.87-74.98) | 74  (34-144) | 38.57  (17.63-74.51) | -0.05  (-0.1-0.01) | |
| India | 105748  (51012-197466) | 31.65  (15.36-58.66) | 277692  (131404-518933) | 40.34  (19.14-75.43) | 0.85  (0.8-0.89) | |
| Indonesia | 11930  (5623-22033) | 14.95  (7.08-27.37) | 20815  (9678-38359) | 14.46  (6.7-26.8) | -0.1  (-0.13--0.07) | |
| Iran (Islamic Republic of) | 3383  (1568-6249) | 17.12  (7.96-31.63) | 8421  (3862-15865) | 16.94  (7.73-31.62) | -0.08  (-0.18-0.02) | |
| Iraq | 888  (427-1676) | 15.38  (7.35-28.71) | 2797  (1306-5227) | 15.06  (7.05-28.09) | -0.07  (-0.1--0.05) | |
| Ireland | 731  (339-1407) | 41.67  (19.27-80.63) | 1276  (591-2433) | 42.38  (19.6-80.64) | 0.06  (0.05-0.07) | |
| Israel | 956  (442-1850) | 40.72  (18.6-78.16) | 1983  (909-3811) | 41.85  (19.17-80.41) | 0.09  (0.08-0.11) | |
| Italy | 12048  (5490-22808) | 35.93  (16.31-67.97) | 12419  (5807-23425) | 34.72  (16.29-65.05) | -0.12  (-0.13--0.11) | |
| Jamaica | 416  (195-781) | 44.87  (21.23-84.56) | 716  (335-1348) | 47  (22.13-88.43) | 0.15  (0.13-0.18) | |
| Japan | 32596  (15052-61755) | 44.96  (20.71-86.19) | 30693  (14411-58028) | 47.03  (21.98-89.82) | 0.15  (0.13-0.18) | |
| Jordan | 177  (83-330) | 15.31  (7.24-28.47) | 783  (367-1466) | 15.56  (7.38-29.13) | 0.06  (0.04-0.07) | |
| Kazakhstan | 4050  (1903-7861) | 51.75  (24.23-99.89) | 5711  (2660-10937) | 54.79  (25.55-105.65) | 0.19  (0.16-0.22) | |
| Kenya | 1831  (860-3392) | 25.89  (12.24-48.01) | 5320  (2505-9861) | 26.32  (12.39-48.88) | 0.06  (0.05-0.06) | |
| Kiribati | 5  (2-9) | 16.03  (7.5-30.19) | 10  (5-18) | 17.56  (8.07-32.21) | 0.31  (0.26-0.36) | |
| Kuwait | 102  (48-195) | 15.81  (7.38-29.72) | 464  (217-877) | 16.06  (7.49-29.98) | 0.06  (0.04-0.07) | |
| Kyrgyzstan | 868  (405-1685) | 49.36  (22.98-95.65) | 1627  (760-3161) | 51.14  (23.87-99.55) | 0.12  (0.03-0.2) | |
| Lao People's Democratic Republic | 262  (124-487) | 16.67  (7.89-31.14) | 624  (289-1146) | 18.27  (8.48-33.54) | 0.32  (0.3-0.34) | |
| Latvia | 1333  (618-2583) | 82.08  (38.11-159.07) | 1052  (481-1971) | 85.77  (39.64-163.45) | 0.16  (0.12-0.19) | |
| Lebanon | 214  (99-397) | 15.34  (7.09-28.48) | 458  (209-873) | 15.53  (7.11-29.5) | 0.04  (0.03-0.06) | |
| Lesotho | 371  (179-694) | 56.36  (27.16-105.61) | 560  (264-1062) | 58.81  (27.7-111.84) | 0.15  (0.14-0.16) | |
| Liberia | 217  (104-407) | 30.86  (14.91-57.39) | 609  (281-1151) | 31.74  (14.61-59.99) | 0.09  (0.08-0.11) | |
| Libya | 193  (92-362) | 15.31  (7.19-28.47) | 590  (277-1092) | 14.99  (7.03-27.65) | -0.08  (-0.1--0.05) | |
| Lithuania | 1173  (543-2247) | 55.02  (25.52-105.42) | 1020  (468-1957) | 57.56  (26.05-110.2) | 0.14  (0.04-0.24) | |
| Luxembourg | 123  (56-237) | 54.21  (24.84-103.99) | 216  (99-415) | 55.28  (25.57-106.74) | 0.05  (0.01-0.1) | |
| Madagascar | 940  (450-1766) | 23.68  (11.3-45.29) | 2410  (1138-4555) | 23.36  (10.93-44.35) | -0.05  (-0.07--0.02) | |
| Malawi | 810  (386-1508) | 25.22  (12.04-47) | 1635  (760-3063) | 24.67  (11.61-46.86) | -0.08  (-0.09--0.06) | |
| Malaysia | 1406  (675-2664) | 18.69  (8.98-35.15) | 3215  (1494-5998) | 20.04  (9.32-37.28) | 0.25  (0.22-0.27) | |
| Maldives | 12  (6-22) | 16.7  (7.89-31.39) | 43  (20-80) | 19.05  (8.81-35.38) | 0.45  (0.43-0.47) | |
| Mali | 853  (417-1557) | 27.99  (13.72-51.3) | 2197  (1043-4135) | 30.86  (14.45-57.95) | 0.33  (0.29-0.37) | |
| Malta | 79  (36-151) | 36.61  (16.54-69.34) | 99  (45-186) | 38.07  (17.21-71.78) | 0.11  (0.06-0.17) | |
| Marshall Islands | 2  (1-4) | 15.07  (6.98-28.06) | 4  (2-8) | 16.46  (7.67-30.42) | 0.3  (0.29-0.32) | |
| Mauritania | 226  (107-426) | 31.33  (14.83-58.8) | 525  (249-995) | 33.46  (15.79-62.04) | 0.21  (0.17-0.26) | |
| Mauritius | 96  (44-179) | 18.03  (8.29-33.45) | 148  (70-281) | 19.25  (8.99-36.51) | 0.22  (0.2-0.25) | |
| Mexico | 18900  (8958-36276) | 59.08  (27.61-112.58) | 38831  (18260-72271) | 56.06  (26.3-104.55) | -0.18  (-0.2--0.17) | |
| Micronesia (Federated States of) | 6  (3-10) | 16.12  (7.67-29.68) | 8  (4-15) | 17.14  (8.24-32.09) | 0.21  (0.19-0.23) | |
| Monaco | 8  (4-16) | 42.46  (19.57-81.93) | 10  (5-19) | 43.07  (19.87-81.76) | 0.05  (0.04-0.05) | |
| Mongolia | 364  (173-704) | 50.52  (23.63-96.06) | 997  (465-1930) | 52.75  (24.71-102.22) | 0.15  (0.12-0.17) | |
| Montenegro | 111  (51-218) | 33.65  (15.4-66.07) | 134  (61-256) | 35.45  (16.19-68.84) | 0.18  (0.16-0.21) | |
| Morocco | 1483  (712-2728) | 14.73  (7.02-27.21) | 2892  (1351-5458) | 14.9  (6.97-28.09) | 0.04  (0.02-0.05) | |
| Mozambique | 1145  (545-2117) | 23.24  (11.03-42.96) | 2549  (1199-4776) | 24.85  (11.64-46.65) | 0.23  (0.22-0.24) | |
| Myanmar | 2813  (1328-5384) | 16.63  (7.85-31.51) | 5570 (2613-10508) | 18.39  (8.62-34.65) | 0.35  (0.33-0.37) | |
| Namibia | 288  (139-541) | 57.88  (27.67-111.54) | 632  (300-1193) | 57.99  (27.42-109.36) | 0.01  (-0.01-0.02) | |
| Nauru | 1  (0-1) | 16.51  (7.77-30.84) | 1  (0-2) | 17.61  (8.27-33.88) | 0.22  (0.2-0.25) | |
| Nepal | 1142  (549-2108) | 15.96  (7.63-29.55) | 2391  (1140-4464) | 15.91  (7.58-29.82) | -0.04  (-0.12-0.05) | |
| Netherlands | 3539  (1621-6748) | 41.43  (19-78.93) | 4266  (1943-7969) | 41.85  (19.12-78.68) | 0.03  (0.02-0.05) | |
| New Zealand | 203  (94-389) | 11.21  (5.16-21.45) | 268  (125-510) | 9.99  (4.67-19) | -0.4  (-0.45--0.35) | |
| Nicaragua | 581  (274-1102) | 47.78  (22.35-90.79) | 1550  (732-2914) | 49.07  (23.2-92.98) | 0.09  (0.08-0.11) | |
| Niger | 701  (334-1298) | 27.99  (13.26-51.62) | 2013  (963-3709) | 29.5  (13.93-54.42) | 0.18  (0.17-0.2) | |
| Nigeria | 9179  (4303-16950) | 31.79  (14.9-59.26) | 27200  (12680-50184) | 33.5  (15.72-62.16) | 0.18  (0.16-0.19) | |
| Niue | 0  (0-0) | 16.65  (7.72-31.62) | 0  (0-0) | 17.89  (8.29-33.4) | 0.25  (0.23-0.27) | |
| North Macedonia | 338  (157-645) | 32.15  (14.97-61.33) | 448  (203-853) | 34.12  (15.75-65.55) | 0.21  (0.19-0.23) | |
| Northern Mariana Islands | 4  (2-7) | 16.62  (7.74-30.96) | 4  (2-8) | 17.48  (8.03-33.25) | 0.18  (0.16-0.19) | |
| Norway | 642  (293-1222) | 27.71  (12.61-52.92) | 855  (388-1645) | 27.85  (12.54-53.23) | -0.02  (-0.08-0.04) | |
| Oman | 75  (36-141) | 14.37  (6.84-26.63) | 262  (120-504) | 15.35  (7.04-29.05) | 0.23  (0.22-0.25) | |
| Pakistan | 9410  (4497-17632) | 25.64  (12.19-48) | 22910  (11041-42685) | 26.28  (12.44-48.57) | 0.08  (0.06-0.11) | |
| Palau | 1  (1-2) | 16.75  (7.8-31.52) | 2  (1-4) | 17.89  (8.21-34.01) | 0.23  (0.21-0.24) | |
| Palestine | 94  (44-176) | 14.5  (6.79-27.43) | 293  (134-549) | 14.61  (6.8-27.45) | 0.02  (0.01-0.04) | |
| Panama | 453  (213-858) | 47.41  (22.39-90.31) | 1052  (482-2009) | 49.69  (22.73-94.75) | 0.16  (0.15-0.17) | |
| Papua New Guinea | 230  (109-426) | 15.28  (7.32-28.5) | 699  (331-1286) | 16.48  (7.79-30.3) | 0.27  (0.24-0.3) | |
| Paraguay | 404  (195-760) | 26.91  (13.03-50.93) | 956  (452-1796) | 28.49  (13.42-53.87) | 0.2  (0.18-0.22) | |
| Peru | 5482  (2645-10396) | 64.85  (31.24-123.48) | 11896 (5660-23007) | 65.91  (31.34-127.31) | 0.06  (0.04-0.08) | |
| Philippines | 5822  (2700-10838) | 23.41  (11.02-43.43) | 13590 (6346-25456) | 25.82  (12.08-48.31) | 0.34  (0.33-0.35) | |
| Poland | 5729  (2646-11121) | 27.25  (12.69-52.46) | 5338 (2522-10122) | 22.99  (10.94-43.12) | -0.59  (-0.64--0.54) | |
| Portugal | 1842  (841-3492) | 32.16  (14.59-60.26) | 2100  (962-3926) | 29.81  (13.7-56.4) | -0.28  (-0.36--0.19) | |
| Puerto Rico | 892  (426-1731) | 46.84  (22.43-90.82) | 1036  (485-1973) | 48.08  (22.53-91.79) | 0.09  (0.08-0.1) | |
| Qatar | 20  (9-39) | 15.43  (7.15-29.03) | 144  (66-270) | 15.51  (7.15-29.09) | 0.02  (-0.01-0.04) | |
| Republic of Korea | 4630  (2139-8836) | 19.65  (9.16-37.47) | 6590 (3070-12585) | 20.98  (9.67-40.79) | 0.22  (0.18-0.25) | |
| Republic of Moldova | 1588  (745-3069) | 65.28  (30.64-126.04) | 1682  (770-3204) | 69.02  (31.74-132.59) | 0.19  (0.17-0.21) | |
| Romania | 3873  (1790-7402) | 30.08  (14.05-57.86) | 3805  (1715-7231) | 31.65  (14.61-61.61) | 0.18  (0.16-0.21) | |
| Russian Federation | 65575  (30604-125617) | 73.69  (33.98-141.83) | 70166  (32508-133968) | 73.12  (33.61-141.63) | -0.02  (-0.04-0) | |
| Rwanda | 590  (281-1107) | 24.45  (11.79-45.68) | 1327  (607-2478) | 24.84  (11.42-46.56) | 0.05  (0.03-0.08) | |
| Saint Kitts and Nevis | 7  (4-14) | 45.73  (21.81-85.6) | 17  (8-32) | 47.12  (21.93-89.5) | 0.151  (0.1-0.11) | |
| Saint Lucia | 23  (11-44) | 44.49  (20.84-84.43) | 49  (23-91) | 45.8  (21.55-85.61) | 0.1  (0.08-0.11) | |
| Saint Vincent and the Grenadines | 17  (8-32) | 43.35  (20.79-80.67) | 28  (13-53) | 45.86  (21.4-86.55) | 0.19  (0.15-0.22) | |
| Samoa | 10  (4-18) | 16.88  (7.87-31.55) | 16  (7-30) | 18.06  (8.39-34.29) | 0.23  (0.21-0.25) | |
| San Marino | 5  (2-10) | 42.14  (19.23-79.69) | 9  (4-18) | 42.71  (19.39-83.05) | 0.05  (0.03-0.07) | |
| Sao Tome and Principe | 12  (6-22) | 30.1  (13.97-56.18) | 27  (13-52) | 31.41  (14.71-59.4) | 0.14  (0.09-0.18) | |
| Saudi Arabia | 674  (316-1275) | 14.26  (6.63-26.87) | 2821  (1320-5471) | 15.19  (7.14-28.95) | 0.22  (0.18-0.25) | |
| Senegal | 800  (391-1474) | 31.83  (15.14-59.04) | 1875  (888-3497) | 32.02  (15.19-59.35) | 0.02  (0-0.03) | |
| Serbia | 1726  (792-3309) | 32.01  (14.68-60.98) | 1761  (805-3368) | 34.16  (15.63-66.37) | 0.23  (0.21-0.24) | |
| Seychelles | 6  (3-10) | 18.24  (8.34-34.48) | 11  (5-21) | 19.27  (8.95-36.57) | 0.19  (0.17-0.21) | |
| Sierra Leone | 412  (199-767) | 30.51  (14.56-57.45) | 1008  (475-1911) | 32.71  (15.45-62.38) | 0.23  (0.2-0.26) | |
| Singapore | 460  (212-872) | 25.41  (11.8-48.31) | 1012  (453-1931) | 27.37  (12.32-52.07) | 0.25  (0.24-0.27) | |
| Slovakia | 1147  (526-2187) | 40.07  (18.39-76.47) | 1445  (664-2746) | 41.77  (19.04-80.29) | 0.14  (0.08-0.2) | |
| Slovenia | 361  (165-690) | 32  (14.6-61.41) | 430  (195-830) | 33.95  (15.61-66.07) | 0.21  (0.16-0.25) | |
| Solomon Islands | 17  (8-31) | 15.38  (7.16-28.84) | 46  (22-88) | 16.74  (7.9-31.64) | 0.29  (0.26-0.32) | |
| Somalia | 597  (283-1100) | 23.61  (11.24-43.81) | 1591  (762-2971) | 23.76  (11.38-44.13) | 0.02  (0-0.04) | |
| South Africa | 10508  (4997-19785) | 67.26  (31.9-126.55) | 19748 (9427-37658) | 65.82  (31.24-125.55) | -0.07  (-0.08--0.07) | |
| South Sudan | 413  (198-760) | 23.39  (11.11-43.23) | 819  (384-1540) | 23.67  (11.23-44.22) | 0.04  (0.03-0.06) | |
| Spain | 11285  (5089-21776) | 53.53  (23.97-102.92) | 16786 (7640-32298) | 54.91  (25.07-105.88) | 0.09  (0.05-0.12) | |
| Sri Lanka | 1404  (661-2642) | 17.39  (8.25-32.83) | 2298  (1058-4275) | 18.35  (8.45-34.06) | 0.19  (0.17-0.2) | |
| Sudan | 894  (422-1684) | 12.61  (6.05-23.46) | 2309  (1077-4320) | 13.78  (6.59-25.41) | 0.31  (0.28-0.33) | |
| Suriname | 74  (35-138) | 45.37  (21.38-85.07) | 152  (73-284) | 47.73  (23.19-89.59) | 0.17  (0.16-0.19) | |
| Sweden | 1505  (694-2855) | 30.67  (14.1-57.77) | 1684  (767-3212) | 29.27  (13.29-56.4) | -0.17  (-0.25--0.08) | |
| Switzerland | 1685  (756-3218) | 41.25  (18.48-79.18) | 2257  (1034-4358) | 41.56  (18.99-79.11) | 0.02  (0.01-0.04) | |
| Syrian Arab Republic | 593  (280-1095) | 14.87  (7.01-27.65) | 1141  (540-2144) | 14.92  (7.08-27.87) | 0.01  (-0.02-0.03) | |
| Taiwan (Province of China) | 1352  (626-2545) | 13.35  (6.23-25.23) | 2298  (1074-4442) | 14.78  (6.91-28.07) | 0.35  (0.33-0.36) | |
| Tajikistan | 859  (406-1663) | 49.1  (22.81-93.74) | 2085  (973-3964) | 49.35  (23.17-93.51) | 0.01  (-0.01-0.03) | |
| Thailand | 4843  (2267-9321) | 17.41  (8.18-33.22) | 8600 (3997-16425) | 18.86  (8.76-35.83) | 0.27  (0.25-0.3) | |
| Timor-Leste | 48  (23-89) | 16.18  (7.52-30.3) | 89  (41-167) | 17.42  (8.12-32.44) | 0.26  (0.24-0.27) | |
| Togo | 365  (174-683) | 29.62  (14.03-54.62) | 1074  (504-2004) | 31.37  (14.59-59.09) | 0.2  (0.18-0.22) | |
| Tokelau | 0  (0-0) | 16.19  (7.71-30.93) | 0  (0-0) | 17.79  (8.29-33.49) | 0.32  (0.28-0.36) | |
| Tonga | 6  (3-11) | 17.03  (8.06-31.8) | 8  (4-16) | 17.97  (8.25-34.03) | 0.19  (0.17-0.21) | |
| Trinidad and Tobago | 241  (115-452) | 45.42  (21.86-84.54) | 389  (185-734) | 47.18  (22.33-89.46) | 0.13  (0.12-0.15) | |
| Tunisia | 493  (231-928) | 14.83  (6.95-28.28) | 1061  (486-2062) | 15.4  (7.05-29.68) | 0.13  (0.08-0.18) | |
| Turkey | 3374  (1606-6194) | 13.67  (6.52-24.99) | 6900 (3132-12894) | 14.48  (6.56-27.01) | 0.2  (0.18-0.22) | |
| Turkmenistan | 668  (313-1290) | 50.67  (23.47-97.19) | 1345  (616-2584) | 52.77  (24.25-100.92) | 0.13  (0.08-0.18) | |
| Tuvalu | 1  (0-1) | 16  (7.53-29.68) | 1  (0-2) | 17.36  (8.1-32.82) | 0.28  (0.26-0.3) | |
| Uganda | 1233  (595-2277) | 23.44  (11.11-43.67) | 3281  (1546-6104) | 23.89  (11.18-44.25) | 0.07  (0.05-0.08) | |
| Ukraine | 22720  (10446-44526) | 71.27  (32.87-140.47) | 21360 (9745-40914) | 71.39  (32.89-137.67) | 0.01  (0-0.02) | |
| United Arab Emirates | 77  (37-146) | 14.77  (6.96-27.21) | 594  (275-1138) | 15.39  (7.09-29.1) | 0.14  (0.12-0.16) | |
| United Kingdom | 11398  (5213-21907) | 35.03  (16.09-67.12) | 12581 (5855-23696) | 31.11  (14.35-58.71) | -0.4  (-0.44--0.36) | |
| United Republic of Tanzania | 2162  (1030-3995) | 25.14  (11.95-46.97) | 5499 (2606-10302) | 25.66  (12.2-48.48) | 0.07  (0.06-0.08) | |
| United States of America | 41967  (19684-79591) | 29.5  (13.87-56.29) | 62893 (29882-117090) | 33.44  (15.7-62.62) | 0.43  (0.36-0.5) | |
| United States Virgin Islands | 27  (13-52) | 45.89  (21.56-87.25) | 30  (14-56) | 47.36  (22.74-89.02) | 0.11  (0.09-0.12) | |
| Uruguay | 514  (236-997) | 31.62  (14.52-61.56) | 671  (306-1270) | 34.17  (15.55-65.66) | 0.27  (0.25-0.29) | |
| Uzbekistan | 3545  (1694-6739) | 48.36  (22.29-91.94) | 8895 (4131-17182) | 52.09  (24.07-99.94) | 0.26  (0.24-0.27) | |
| Vanuatu | 8  (4-16) | 15.69  (7.49-29.3) | 21  (10-40) | 16.98  (8-32) | 0.27  (0.25-0.29) | |
| Venezuela (Bolivarian Republic of) | 3768  (1786-7193) | 51.14  (23.82-96.27) | 8133 (3794-15164) | 50.74  (23.77-94.93) | -0.03  (-0.04--0.02) | |
| Viet Nam | 4275  (1998-8151) | 15.43  (7.19-28.84) | 10121 (4620-18930) | 17.56  (8.04-32.9) | 0.45  (0.43-0.47) | |
| Yemen | 577  (281-1067) | 13.78  (6.76-25.27) | 1779  (856-3287) | 14.24  (6.89-26.37) | 0.11  (0.1-0.13) | |
| Zambia | 639  (305-1193) | 25.84  (12.21-48.86) | 1695  (779-3196) | 25.85  (12.01-48.31) | 0  (-0.01-0.01) | |
| Zimbabwe | 1978  (951-3749) | 59.36  (28.51-112.19) | 3654  (1739-6882) | 58.44  (27.81-110.36) | -0.05  (-0.08--0.02) | |

**Notes:**

YLDs: years lived with disability; ASR: age-standardized rate.

ASR for YLDs is computed by direct standardization with global standard population in GBD 2019.

Net drifts are estimates derived from the age-period-cohort model and denotes overall annual percent change in YLDs rate.

Parenthesis for all GBD health estimate indicates 95% uncertainty intervals; parenthesis for net drift indicates 95% confidence intervals.

**Supplementary Figures**


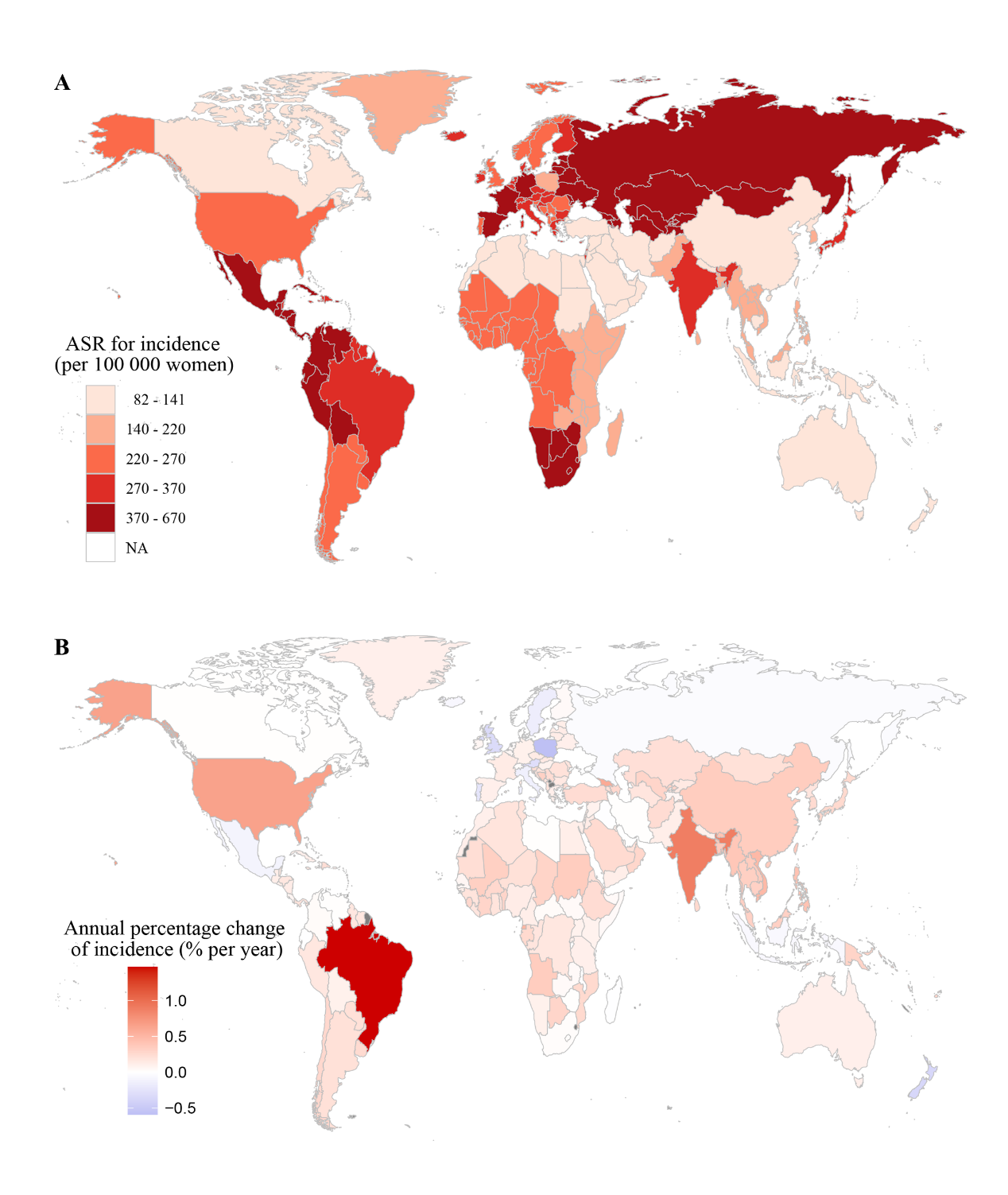


**Figure S1.** The global distribution of age-standardized rate for incidence in 2019 (A) and net drift of incidence rate during 1990−2019 (B) for uterine fibroids in 204 countries and territories.


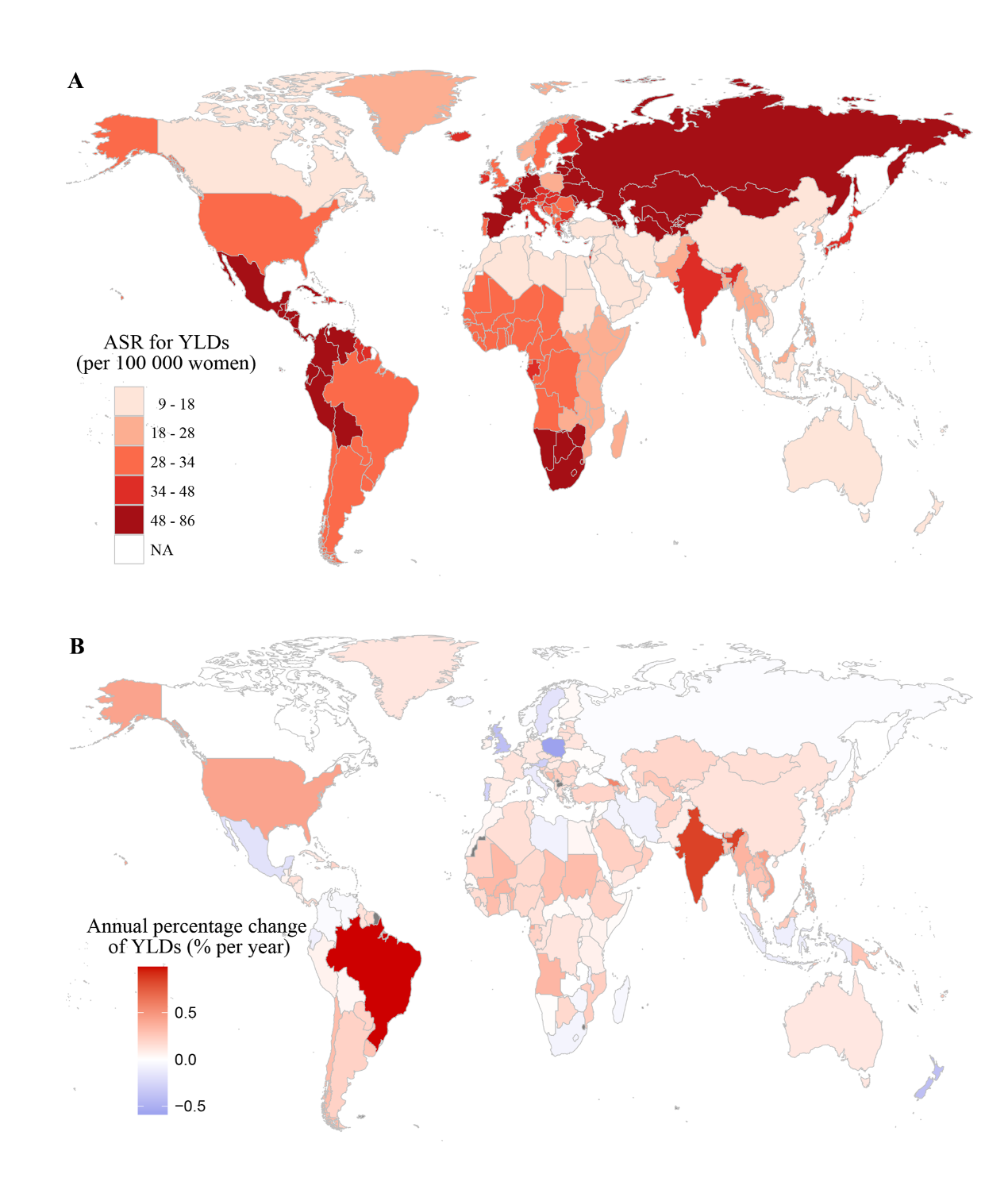


**Figure S2.** The global distribution of age-standardized rate for years lived with disability in 2019 (A) and net drift of years lived with disability rate during 1990−2019 (B) for uterine fibroids in 204 countries and territories.
